# *In vitro* properties of patient serum predict clinical outcome after high dose rate brachytherapy of hepatocellular carcinoma

**DOI:** 10.1002/1878-0261.70122

**Published:** 2025-09-12

**Authors:** Lukas Salvermoser, Jan Niklas Schäfer, Shraga Nahum Goldberg, Philipp Maximilian Kazmierczak, Moritz Nikolaus Gröper, Philipp Franz Linden, Elif Öcal, Tanja Burkard, Stefanie Corradini, Najib Ben Khaled, Moritz Wildgruber, Max Seidensticker, Jens Ricke, Matthias Stechele, Marianna Alunni‐Fabbroni

**Affiliations:** ^1^ Department of Radiology University Hospital, LMU Munich Germany; ^2^ Goldyne Savad Institute of Gene Therapy and Division of Image‐Guided Therapy and Interventional Oncology, Department of Radiology Hadassah Hebrew University Medical Center Jerusalem Israel; ^3^ Department of Radiation Oncology University Hospital, LMU Munich Germany; ^4^ Department of Medicine II University Hospital, LMU Munich Germany

**Keywords:** BrdU incorporation, hepatocellular carcinoma, hepatoma cell lines, high dose rate brachytherapy, local tumor ablation, serum incubation

## Abstract

Tumor recurrence after local tumor ablation, including high dose rate brachytherapy (HDR‐BT), represents a substantial challenge in hepatocellular carcinoma (HCC) treatment. This study aimed to investigate whether induced factors that appear in patient serum after HDR‐BT alter HCC growth *in vitro*, and whether this correlates with outcome. In total, 23 HCC patients [Barcelona clinic liver cancer (BCLC) stage A or B] were treated by HDR‐BT (1 × 15 Gy) and classified as responders in case of no progression within 6 months and no diffuse systemic progression within 2 years (*n* = 12), or nonresponders with recurrence within 6 months and/or diffuse systemic tumor progression or extrahepatic disease within 2 years (*n* = 11). Patient serum was obtained at baseline and 48 h postprocedure. Two hepatoma cell lines (Huh7, HepG2) were incubated for 72 h in the presence of 20% serum. BrdU incorporation was assessed for serum incubation at baseline and 48 h post‐HDR‐BT. BrdU incorporation post‐HDR‐BT compared to baseline was significantly elevated in nonresponders compared to responders for both Huh7 and HepG2. Likewise, confirmatory Cyclin E studies revealed different induction kinetics between a subset of representative responders and nonresponders in HepG2. Time to systemic progression (TTSP) in patients with increased BrdU incorporation was significantly shorter compared to patients with decreased BrdU incorporation after serum incubation. These data indicate that poor outcome following HDR‐BT is associated with increased measurable proliferation parameters of hepatoma cell lines *in vitro* after exposure to patient serum, offering insights into post‐treatment tumor biology and a potential biomarker of clinical outcome.

## Graphical abstract

Following high dose rate brachytherapy (HDR‐BT) for hepatocellular carcinoma (HCC), patients were classified as responders and nonresponders. Post‐therapy serum induced increased BrdU incorporation and Cyclin E expression of Huh7 and HepG2 cells in nonresponders, but decreased levels in responders. High BrdU incorporation was associated with shorter time to systemic progression (TTSP) and elevated plasma levels of PTN and CRTAM.

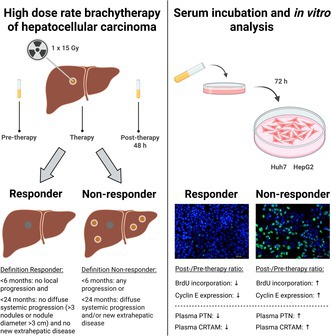

**Abbreviations**
AUC: area under the curve
BCLC: Barcelona clinic liver cancer
BrdU: 5‐bromo‐2′‐deoxyuridine
CRTAM: cytotoxic and regulatory T cell molecule
CT: computed tomography
DAPI: 4′,6‐diamidino‐2‐phenylindole
FCS: Fetal calf serum
Gd‐EOB‐DTPA: Gadolinium‐ethoxybenzyl‐diethylenetriamine pentaacetic acid
HCC: hepatocellular carcinoma
HDR‐BT: high dose rate brachytherapy
HPF: high‐power field
MRI: magnetic resonance imaging
NPX: normalized protein expression
PBS: phosphate‐buffered saline
PTN: pleiotrophin
ROC: receiver operating characteristics
TTSP: time to systemic progression

## 1 Introduction

Despite successful integration into current treatment guidelines for hepatocellular carcinoma (HCC), recurrence after local tumor ablation, such as high dose rate brachytherapy (HDR‐BT), represents a substantial drawback and challenge in clinical practice. Potential explanations for these findings are mostly based on investigations of thermal techniques and include unwanted, secondary effects caused by the induction of different cellular and molecular alterations [1].

On an epigenetic level, alterations of microRNAs can either suppress [2] or facilitate tumor growth in HCC [3]. Intra‐ and extracellular protein alteration can enhance tumor growth, as shown by increased HCC tumorigenesis in Smad7‐KO mice via STAT3 [4]. Also, following thermal ablation, STAT3 acts as a central mediator in the pro‐oncogenic cascade after hepatic radiofrequency ablation [5]. As most data on tumorigenic effects following local ablation focuses on thermal techniques, there are only limited data on systemic effects following radiation‐based procedures. It has previously been shown that circulating post‐therapeutic growth factors such as increased EGF and angiogenic proteins including angiopoietin‐1 may be associated with patient outcome [6]. However, this promising clinical association does not sufficiently prove direct effects of post‐therapeutic serologic growth factors on tumor cell growth.

Specifically, the direct effect of pre‐ and post‐therapeutic patient serum on tumor cell growth *in vitro* following HDR‐BT has not yet been shown. We hypothesize that the serum of patients with poor outcomes promotes tumor cell growth compared to patients with beneficial outcomes after incubation *in vitro*. Accordingly, the purpose of this study was to evaluate alterations in parameters of cell proliferation and cell cycle kinetics in two different hepatoma cell lines exposed to patient serum before and after local tumor ablation and correlate results with clinical outcomes.

## 2 Materials and methods

### 2.1 Study population and ethical considerations

For this prospective study, 23 patients with previously untreated HCC with Barcelona clinic liver cancer (BCLC) stage A or B were included. Patients presented at the Department of Radiology at LMU University Hospital, Munich, Germany, between August 2017 and November 2019. Ethics approval was provided by the ethics committee ‘Ethikkommission bei der LMU München’ (reference number: 17‐346) on June 20, 2017, and August 26, 2020. The ‘ESTIMATE’ trial was registered at the ‘Deutsches Register Klinischer Studien’ (reference number: DRKS00010587). All patients provided written informed consent for local ablative treatment and study inclusion. The study was conducted in accordance with the Declaration of Helsinki.

### 2.2 Study procedures

HDR‐BT was performed as previously described [3]. Briefly, brachytherapy catheter placement under CT fluoroscopic guidance (Somatom Edge, Siemens Healthineers, Forchheim, Germany) was followed by administration of a dose of 15 Gy in a single fraction using an afterloading system (Nucletron, Elekta Ab, Stockholm, Sweden) with an Iridium‐192 source.

Twenty‐four hours before and 48 h after HDR‐BT, all patients underwent a peripheral blood draw of 5 mL collected in S‐Monovette EDTA tubes and Monovette EDTA tubes (Sarstedt, Nümbrecht, Germany). Within 60 min after blood collection, serum and plasma were obtained by centrifugation at 1000 ***g*** for 5 min at 4 °C. Serum was aliquoted and stored at −80 °C until use.

Baseline imaging was performed on an average of 10 days before HDR‐BT, including MRI of the liver with hepatocyte‐specific Gd‐EOB‐DTPA contrast agent (Primovist, Bayer Vital, Leverkusen, Germany) and/or contrast‐enhanced CT of the chest, abdomen, and pelvis. Follow‐up imaging was carried out in three‐month intervals, including MRI and CT scans as described above.

### 2.3 Oncological response and outcome

Stratification of the cohort into responders and nonresponders was performed as previously described [3]. Briefly, responders demonstrated no limited or diffuse systemic tumor progression within 6 months following HDR‐BT and showed no diffuse systemic progression (more than 3 nodules or at least one nodule with a diameter of > 3 cm) within 6 to 24 months after HDR‐BT. Nonresponders demonstrated tumor recurrence within 6 months after HDR‐BT and/or (a) tumor progression of more than 3 nodules, (b) at least one nodule with a diameter > 3 cm, or (c) extrahepatic tumor manifestation within 24 months following the procedure. Time to systemic progression (TTSP) was defined as the interval between HDR‐BT and the occurrence of any of the criteria mentioned above. Liver transplantation was performed in two patients 9 months after HDR‐BT. For these two cases, MRI and CT imaging demonstrated no tumor recurrence until that time, and pathological examination of the liver explant did not detect any evidence of viable tumor. Hence, these patients were stratified as responders.

### 2.4 Cell culture

The human hepatocellular carcinoma cell line Huh7 (CVCL_0336) was maintained in RPMI 1640 (Merck, Darmstadt, Germany) supplemented with 10% FCS (Merck), 1% l‐glutamine (PAN Biotech, Aidenbach, Germany), and 1% Penicillin–Streptomycin (Thermo Fisher Scientific, Waltham, MA, USA). The human hepatocellular carcinoma cell line HepG2 (CVCL_0027) was maintained in Low Glucose Dulbecco's Modified Eagle Medium (Merck) supplemented with 10% FCS, 1% l‐glutamine (PAN Biotech), and 1% Penicillin–Streptomycin (Thermo Fisher Scientific). Both cell lines were incubated in a 5% CO_2_ humidified atmosphere at 37 °C. For each cell line, cell line authentication has been performed via DNA profiling of 17 different and highly polymorphic Short Tandem Repeat loci showing a complete corresponding match to the reference Short Tandem Repeat profiles. Testing for mycoplasma contamination was negative. Cell lines were kindly provided from frozen stock sources from the Goldyne Savad Institute of Gene Therapy at Hadassah Hebrew University Medical Center, Jerusalem. Huh7 was acquired from JRCB (Tokyo, Japan), and HepG2 from ATCC (Manassas, VA, USA).

### 2.5 Serum incubation and BrdU incorporation assay

Experiments were conducted on the two hepatoma cell lines Huh7 and HepG2. Prior to incubation with human serum, cells were plated on 8‐well chambers (Sarstedt, Nümbrecht, Germany) at a density of 40 000 cells per well and incubated with regular medium. 24 h after plating, regular medium was replaced with fresh medium containing 20% patient serum. Serum incubation took place for 72 h at 37 °C at 5% CO_2_. Cell proliferation was assessed by measuring the 5‐bromo‐2′‐deoxyuridine (BrdU) incorporation by hepatoma cells after serum incubation using the BrdU Cell Proliferation Kit (Sigma‐Aldrich, St. Louis, MO, USA) according to the manufacturer's protocol. Nuclei were counterstained with DAPI (ThermoFisher Scientific, Waltham, MA, USA). As a final step, slides were covered with Fluoromount‐G (Biozol, Eching, Germany) and stored in the dark.

### 2.6 Immunohistochemical analysis of cyclin E expression

HepG2 and Huh7 cells were plated in 8‐well chambers at a density of 40 000 cells per well and incubated in regular medium for 24 h. To perform cell cycle arrest by nutrient starvation, regular medium was then replaced by a fetal calf serum (FCS)‐deprived medium for 12 h. To induce cell cycle activity, FCS‐deprived medium was replaced by fresh medium containing 20% patient's serum. Serum was used from three representative responders that showed a BrdU incorporation ratio < 0.9 in both cell lines (Patients 8, 9, 12) and three representative nonresponders that showed a BrdU incorporation ratio > 1.1 in both cell lines (patients 13, 21, 22). Immunohistochemical staining (IHC) was performed to measure Cyclin E expression after incubation periods of 3, 6, 9, 11, and 13 h. The measured time points were selected based upon literature reports of a Cyclin E peak expression at 6 to 9 h after cell cycle induction [7, 8, 9]. Briefly, cells were permeabilized for 5 min in cold methanol (Merck), washed with phosphate‐buffered saline (PBS) (Merck) three times for 10 min, and incubated with UltraCruz blocking reagent (Santa Cruz Biotechnology, Dallas, TX, USA) for 30 min at room temperature. Cells were then incubated overnight with a mouse monoclonal anti‐human Cyclin E antibody (clone HE 11, dilution 1 : 25, Santa Cruz Biotechnology) directly labeled with Alexa Fluor 594 at 4 °C. After five washing steps with PBS, DAPI (1 : 10 000) staining was performed and slides were mounted as described above.

### 2.7 Image acquisition and analysis

Image acquisition was performed using a fluorescence microscope (Leica DM 2500 LED, Leica Microsystems, Wetzlar, Germany) with protection from direct light exposure using the software leica application suite, version 4.13 (Leica Microsystems, Wetzlar, Germany). Five high‐power fields (HPFs) within areas with a homogenous cell distribution in the DAPI staining were randomly selected for analysis for each sample using a 20× magnification. The area fraction of DAPI, BrdU, and Cyclin E‐positive cells in relation to the total area of the respective HPF was evaluated using the software imagej, version 1.54j (NIH, Bethesda, MD, USA). After that, a BrdU/DAPI or Cyclin E/DAPI was used. Using ImageJ, a consistent color threshold per staining type was selected to ensure consistent sensitivity for each HPF. The conversion of the stained and fluorescent areas within the HPF was performed using a binary color function.

BrdU and Cyclin E quotients were evaluated for each HPF to reflect the proportion of cells actively synthesizing DNA (BrdU‐positive) or expressing Cyclin E (Cyclin E‐positive) in comparison to all detected cells (DAPI‐positive). To quantify BrdU incorporation, a BrdU/DAPI quotient for each HPF was calculated as the fraction of the HPF with a positive BrdU staining signal divided by the fraction of the HPF with a positive DAPI staining according to the following formula: (% of the area with positive BrdU signal)/(% of the area with positive DAPI signal). Likewise, Cyclin E expression was evaluated with the fraction of the HPF with a positive Cyclin E staining signal divided by the fraction of the HPF with a positive DAPI staining signal using the formula: (% of the area with positive Cyclin E signal)/(% of the area with positive DAPI signal).

To assess the effect of HDR‐BT on cell BrdU incorporation during serum incubation, the BrdU/DAPI quotient of cells incubated with serum obtained 48 h after HDR‐BT (% BrdU post) was divided by the BrdU/DAPI quotient of cells incubated with serum obtained 24 h before HDR‐BT (% BrdU pre). Accordingly, the BrdU incorporation ratio ‘% BrdU (post/pre)’ was calculated using the following formula: (% BrdU post)/(% BrdU pre). Consequently, the experiments of each cell line rendered an individual BrdU incorporation ratio for each patient. Hence, a ratio < 1 indicated decreased cell BrdU incorporation by incubation with serum obtained after HDR‐BT compared to baseline serum obtained before HDR‐BT. By contrast, a ratio > 1 indicated increased cell BrdU incorporation by incubation with serum obtained after HDR‐BT compared to baseline serum obtained before HDR‐BT. Cyclin E ratios were evaluated by the Cyclin E/DAPI quotient resulting from the experiments performed with serum obtained 48 h after HDR‐BT (Cyclin E/DAPI post) divided by the Cyclin E/DAPI quotient resulting from the experiments with serum obtained 24 h before HDR‐BT (Cyclin E/DAPI pre). Accordingly, the Cyclin E expression ratio was calculated using the following formula: (Cyclin E/DAPI post)/(Cyclin E/DAPI pre). Hence, a ratio < 1 indicated decreased Cyclin E expression, and a ratio > 1 indicated increased Cyclin E expression after HDR‐BT in comparison to baseline before therapy.

### 2.8 Proximity extension assay of plasma proteins

For each patient plasma sample 24 h before and 48 h after HDR‐BT was analyzed with proximity extension assay as previously described [6]. Briefly, analysis was performed for 92 proteins of the Olink proteomics Target 96 Immuno‐Oncology panel (Olink Proteomics, Uppsala, Sweden). Normalized protein expression (NPX) was used to display protein levels on a log2 scale.

### 2.9 Statistical analysis

Baseline clinical and technical patient characteristics and baseline BrdU incorporation between responders and nonresponders were compared using the Mann–Whitney *U* test. BrdU incorporation ratios were evaluated by the BrdU incorporation from serum obtained after HDR‐BT divided by the BrdU incorporation from serum obtained before HDR‐BT. For each cell line, mean BrdU incorporation ratios < 0.9 indicated a decrease, while ratios > 1.1 indicated an increase. This approach has previously been applied successfully as it has the advantage that nearly unchanged BrdU incorporations are not further interpreted [6]. The ratios with substantial differences were subject to all subsequent analysis steps. BrdU incorporation ratios between 0.9 and 1.1 were considered unchanged. To analyze the relation between the BrdU incorporation ratio (< 0.9; > 1.1) and patient response (responder; nonresponder) to HDR‐BT, contingency tables were created and the groups based on the BrdU incorporation ratio (< 0.9; > 1.1) and response (responder; nonresponder). Fisher's exact test was performed to assess statistically significant differences. The Mann–Whitney *U* test was used to compare the group of responders (*n* = 12) with the group of nonresponders (*n* = 11) for the individual BrdU incorporation ratios. The area under the curve (AUC) was calculated based on the BrdU incorporation ratio of all patients (including ratios of 0.9–1.1) stratified into responders (positives) and nonresponders (negatives). Stated cut‐off values were determined by applying the Youden Index using the formula Youden's *J* = Sensitivity + Specificity − 1. Survival analysis compared the TTSP of patients with a BrdU incorporation ratio < 0.9 and > 1.1 using the log‐rank test. Similar to BrdU incorporation ratios, Cyclin E ratios were evaluated by the Cyclin E from serum post‐HDR‐BT divided by the Cyclin E from serum pre‐HDR‐BT. Comparison of Cyclin E ratios between responders and nonresponders was performed using a spline linear mixed model (Toeplitz covariance structure, knots at time points 6 and 9 h) with the Cyclin E ratio as a dependent variable and the effects of response, time, and interaction of time and response. To compare protein values pre‐ and post‐therapy, ΔNPX values were obtained by subtracting the pretherapy NPX values from the post‐therapy NPX values. Subsequently, the power of two from each absolute ΔNPX resulted in a Post/Pre protein ratio. To evaluate statistical correlation, Spearman correlation was performed between Post/Pre protein ratio and BrdU incorporation ratio for Huh7 and HepG2, respectively. The proteins with the strongest correlation between Post/Pre protein ratio and a BrdU incorporation ratio were considered for further analysis (all proteins with a Spearman *r* of > 0.5 from the mean of Huh7 and HepG2). Ultimately, patient protein values were stratified according to the BrdU incorporation of Huh7 and HepG2 (< 1.0; > 1.0), and groups were compared with the Mann–Whitney *U* test. For visualization in the heatmap, BrdU incorporation ratios and protein levels underwent log2‐transformation and z‐score normalization.

Statistical analysis was performed with graphpad prism, version 10.2.3 (GraphPad, San Diego, CA, USA) and sas, version 9.4 (SAS Institute, Cary, NC, USA).

## 3 Results

### 3.1 Study population and characteristics

The cohort consisted of a total of 23 patients (Table S1). Out of these, 19 were male and four were female. The most common underlying etiology for HCC development was alcohol‐induced (*n* = 8, 34.8%), followed by Hepatitis C and cryptogenic origin (both *n* = 6, 26.1%, respectively). As for the severity of liver cirrhosis, the majority of patients were classified as Child‐Pugh A (*n* = 18, 78.3%).

Regarding clinical outcome, the cohort comprised 12 responders (52.2%) and 11 nonresponders (47.8%). For all obtained baseline characteristics, no significant difference between responders and nonresponders was observed (Tables S1 and S2). The average time of follow‐up was 628 days (183–1224 days). The average TTSP or loss of follow‐up was 892 days for responders and 341 days for nonresponders.

### 3.2 BrdU incorporation of HCC cells exposed to patient serum correlates with therapy response

**Fig. 1:**
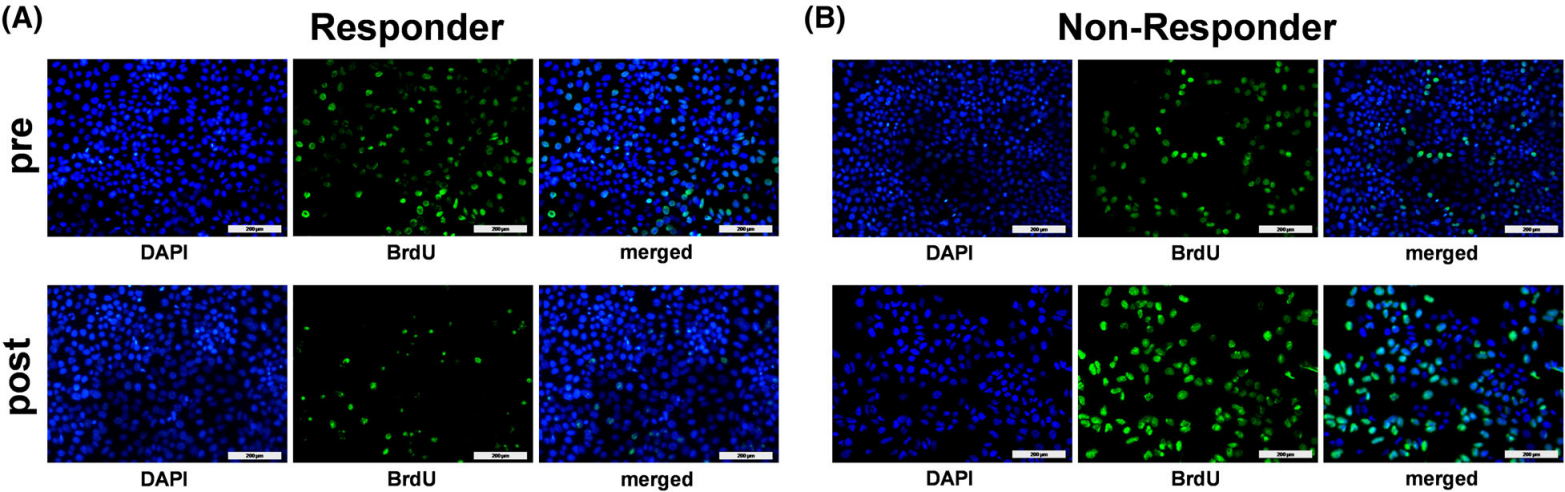
BrdU incorporation of two representative patients in Huh7. Upper panel: High‐power fields (HPFs) (20× magnification) of DAPI, BrdU staining, and the merged image before high dose rate brachytherapy (HDR‐BT) (pre); Lower panel: HPFs after HDR‐BT (post). Responder (A): patient 9, decreasing BrdU incorporation following HDR‐BT compared to baseline. Nonresponder (B): patient 15, increasing BrdU incorporation following HDR‐BT compared to baseline. Much greater BrdU incorporation after HDR‐BT was observed in the nonresponder (pre: 26.65%, post: 78.04%), compared to the responder (pre: 29.75%, post: 5.76%). Scale bars represent 200 μm.

Figure 1 displays representative high‐power fields (HPFs, 20× magnification) of a patient with poor and positive responses to therapy with respective levels of BrdU Incorporation.

**Fig. 2:**
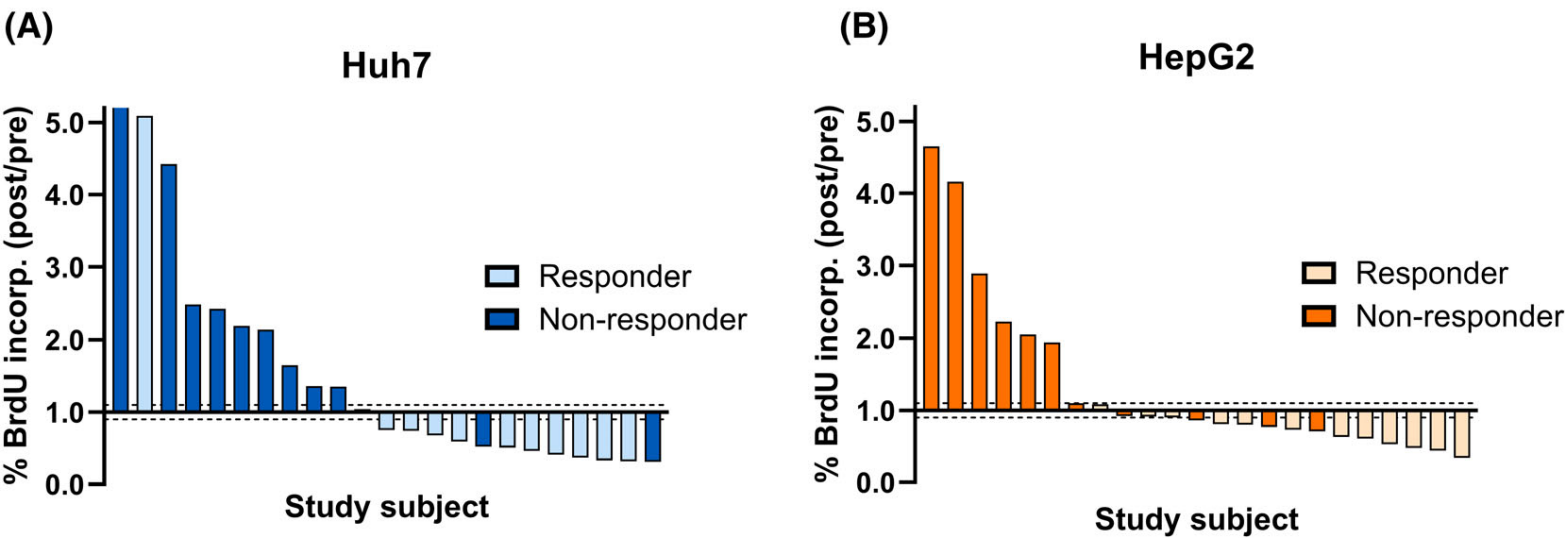
BrdU incorporation after serum incubation per individual study subject. Waterfall plots of all BrdU incorporation ratios per individual study subject according to clinical outcome for both cell lines Huh7 (A) and HepG2 (B). Nonresponders are marked in dark blue and dark orange for each cell line, respectively; responders are marked in light blue and light orange for each cell line, respectively. For each cell line, two independent experiments were performed to assess BrdU incorporation.

**Fig. 3:**
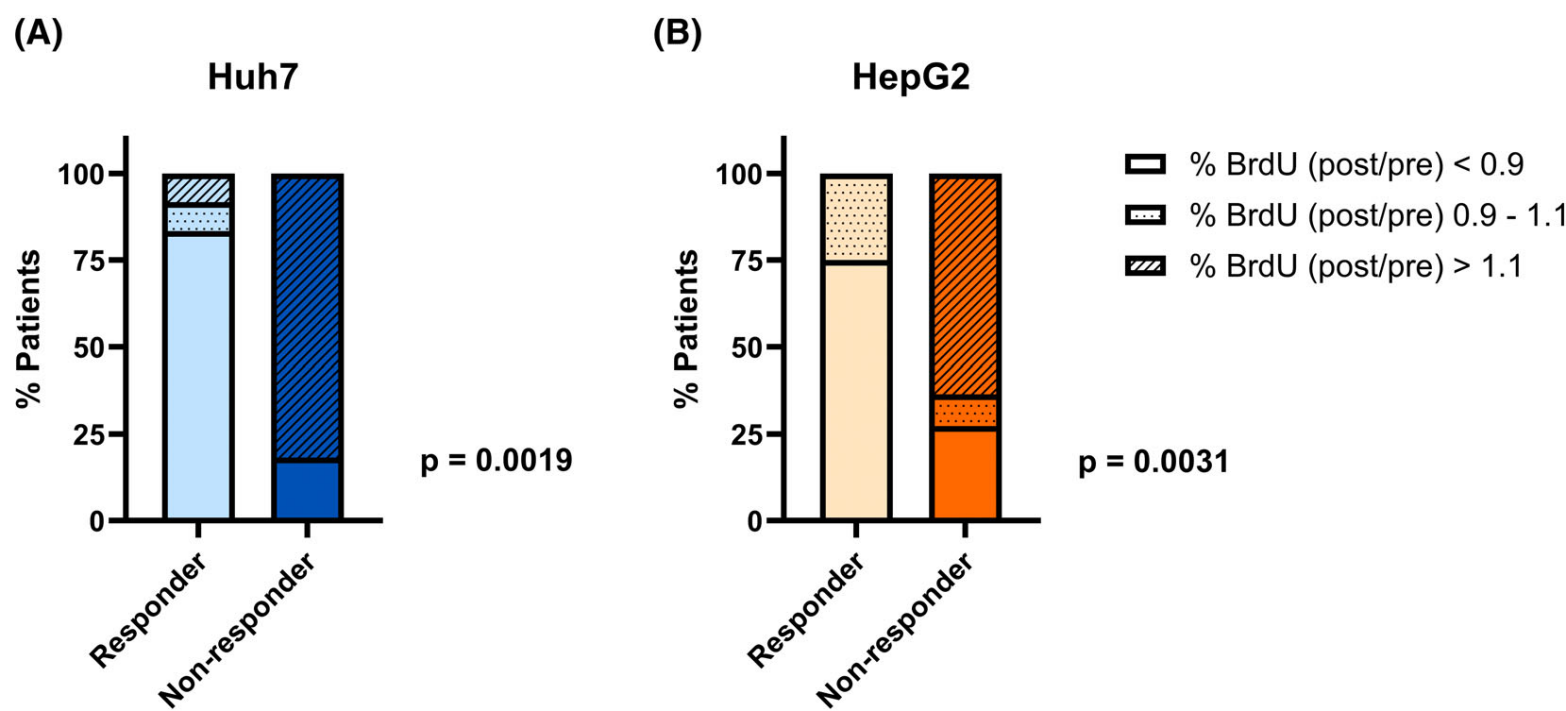
BrdU incorporation after serum incubation in responders and nonresponders. Stacked bar plots show significantly different fractions of responders and nonresponders with substantially increased (> 1.1; hatched) or decreased (< 0.9; unfilled) as well as not substantially changed (0.9–1.1; dotted) BrdU incorporation ratios for Huh7 (A) and HepG2 (B). For each cell line, two independent experiments were performed to assess BrdU incorporation. Fisher's exact test was used to determine statistical significance.

For Huh7, 22 of 23 patients (95.7%) showed substantial differences of either < 0.9 or > 1.1 BrdU incorporation after HDR‐BT compared to baseline, while 1 patient (4.3%) was not showing differences beyond this margin of error. Eleven patients with substantial differences of BrdU incorporation ratio were nonresponders, of which nine (81.8%) showed increasing, and two (18.2%) showed decreasing values. The remaining 11 patients with substantial differences in BrdU incorporation were responders, of which 10 (90.9%) showed decreasing values, and 1 (9.1%) showed increased BrdU incorporation ratio (Figs 2A and 3A, *P* = 0.0019; Fig. S1A).

BrdU incorporation from baseline serum did not differ significantly between responders and nonresponders (median % BrdU incorporation: responders 29.8 [22.9–40.1], nonresponders 23.1 [11.8–29.2]; Table 1, *P* = 0.1179). From baseline, the post‐treatment values decreased to 17.5 [14.7–20.7] in responders, whereas in nonresponders, post‐treatment values increased to 28.0 [15.1–41.5] (Table 1). Statistical analysis of the respective BrdU incorporation ratio revealed significantly increased ratios in nonresponders in comparison to responders (responders: 0.51 [0.39–0.71], nonresponders: 2.14 [1.35–2.46], Fig. S2A, *P* = 0.0158). Table S3A,B represent the corresponding absolute BrdU incorporation.

**Table 1:** BrdU incorporation after serum incubation of Huh7. Indicated is the BrdU incorporation before (pre‐therapy) and after HDR‐BT (post‐therapy), as well as the ratio created from both time points (Ratio post/pre) for each patient.

Patient ID	Response	Experiment 1			Experiment 2			Combined		
		Pre therapy	Post therapy	Ratio post/pre	Pre therapy	Post therapy	Ratio post/pre	Pre therapy	Post therapy	Ratio post/pre
1	Responder	6.20	2.60	0.42	27.90	30.10	1.08	17.05	16.35	0.75
2	Responder	11.30	9.80	0.87	29.70	18.10	0.61	20.50	13.95	0.74
3	Responder	16.60	5.60	0.34	46.20	18.40	0.40	31.40	12.00	0.37
4	Responder	27.60	12.90	0.47	49.70	22.80	0.46	38.65	17.85	0.46
5	Responder	2.20	16.30	7.41	14.40	40.00	2.78	8.30	28.15	5.09
6	Responder	33.60	23.90	0.71	34.20	15.90	0.46	33.90	19.90	0.59
7	Responder	28.40	11.40	0.40	19.10	18.40	0.96	23.75	14.90	0.68
8	Responder	31.50	9.50	0.30	18.40	13.20	0.72	24.95	11.35	0.51
9	Responder	32.80	3.30	0.10	55.70	30.90	0.55	44.25	17.10	0.33
10	Responder	40.90	9.00	0.22	73.40	30.20	0.41	57.15	19.60	0.32
11	Responder	11.20	12.90	1.15	45.10	41.80	0.93	28.15	27.35	1.04
12	Responder	22.50	7.00	0.31	77.10	39.20	0.51	49.80	23.10	0.41
13	Nonresponder	2.50	18.00	7.20	12.50	20.40	1.63	7.50	19.20	4.42
14	Nonresponder	7.30	13.20	1.81	13.50	42.70	3.16	10.40	27.95	2.49
15	Nonresponder	4.20	5.60	1.33	5.90	20.80	3.53	5.05	13.20	2.43
16	Nonresponder	38.00	14.40	0.38	25.40	16.90	0.67	31.70	15.65	0.52
17	Nonresponder	9.20	21.50	2.34	17.30	35.20	2.03	13.25	28.35	2.19
18	Nonresponder	29.00	7.80	0.27	54.10	18.90	0.35	41.55	13.35	0.31
19	Nonresponder	26.40	28.00	1.06	24.20	40.00	1.65	25.30	34.00	1.36
20	Nonresponder	36.10	2.70	0.07	10.00	26.20	2.62	23.05	14.45	1.35
21	Nonresponder	11.90	25.60	2.15	63.00	72.30	1.15	37.45	48.95	1.65
22	Nonresponder	17.40	45.90	2.64	36.10	59.20	1.64	26.75	52.55	2.14
23	Nonresponder	4.60	59.50	12.93	25.40	96.90	3.81	15.00	78.20	8.37

For HepG2, 19 of 23 patients (82.6%) showed substantial differences of either < 0.9 or > 1.1 BrdU incorporation post compared to pre‐HDR‐BT, while four patients (17.4%) did not demonstrate differences beyond the margin of error. Ten patients with substantial differences in their BrdU incorporation ratio were nonresponders, of which seven (70.0%) showed increasing and three (30.0%) showed decreasing values. The remaining nine patients with substantial differences in BrdU incorporation were responders, of which all nine (100.0%) showed decreasing BrdU incorporation ratio values (Figs 2B and 3B, *P* = 0.0031; Fig. S1B).

BrdU incorporation from baseline serum did not differ significantly between responders and nonresponders (median % BrdU incorporation: responders 14.0 [9.9–18.8], nonresponders 12.7 [9.1–15.3]; Table 2, *P* = 0.4400). Post‐treatment values decreased to 10.8 [8.1–14.1] in responders and increased to 14.3 [11.2–16.7] in nonresponders (Table 2). Statistical analysis of the respective BrdU incorporation ratio revealed significantly increased ratios in nonresponders in comparison with responders (responders 0.61 [0.48–0.73], nonresponders 2.00 [0.92–2.72], Fig. S2B, *P* = 0.0004).

**Table 2:** BrdU incorporation after serum incubation of HepG2. Indicated is the BrdU incorporation before (pre‐therapy) and after HDR‐BT (post‐therapy), as well as the ratio created from both time points (Ratio post/pre) for each patient.

Patient ID	Response	Experiment 1			Experiment 2			Combined		
		Pre therapy	Post therapy	Ratio post/pre	Pre therapy	Post therapy	Ratio post/pre	Pre therapy	Post therapy	Ratio post/pre
1	Responder	47.05	38.07	0.81	9.20	6.00	0.65	28.12	22.04	0.73
2	Responder	50.26	46.44	0.92	18.40	12.30	0.67	34.33	29.37	0.80
3	Responder	11.45	5.67	0.50	1.60	0.60	0.38	6.53	3.13	0.44
4	Responder	16.84	13.69	0.81	6.77	6.76	1.00	11.80	10.22	0.91
5	Responder	13.15	25.93	1.97	1.60	0.30	0.19	7.38	13.12	1.08
6	Responder	24.63	24.36	0.99	6.20	0.40	0.06	15.41	12.38	0.53
7	Responder	19.59	11.92	0.61	1.40	0.50	0.36	10.49	6.21	0.48
8	Responder	16.34	13.95	0.85	8.80	3.60	0.41	12.57	8.77	0.63
9	Responder	15.17	9.29	0.61	0.91	0.91	1.00	8.04	5.10	0.81
10	Responder	30.45	21.64	0.71	0.80	0.40	0.50	15.63	11.02	0.61
11	Responder	30.66	31.17	1.02	3.80	3.00	0.79	17.23	17.08	0.90
12	Responder	43.44	20.20	0.47	3.60	0.80	0.22	23.52	10.50	0.34
13	Nonresponder	29.28	33.33	1.14	0.60	4.90	8.17	14.94	19.12	4.65
14	Nonresponder	19.32	31.66	1.64	1.60	3.60	2.25	10.46	17.63	1.94
15	Nonresponder	13.45	20.19	1.50	12.00	8.40	0.70	12.73	14.30	1.10
16	Nonresponder	42.40	29.26	0.69	3.90	4.50	1.15	23.15	16.88	0.92
17	Nonresponder	23.06	28.23	1.22	9.50	4.80	0.51	16.28	16.51	0.86
18	Nonresponder	16.49	12.32	0.75	11.70	7.90	0.68	14.10	10.11	0.71
19	Nonresponder	27.91	27.62	0.99	3.40	1.90	0.56	15.65	14.76	0.77
20	Nonresponder	20.87	6.69	0.32	0.90	7.20	8.00	10.88	6.94	4.16
21	Nonresponder	14.24	21.07	1.48	1.30	3.40	2.62	7.77	12.24	2.05
22	Nonresponder	5.24	7.06	1.35	0.90	2.80	3.11	3.07	4.93	2.23
23	Nonresponder	12.51	24.72	1.98	0.50	1.90	3.80	6.51	13.31	2.89

In 20 of 23 patients (86.9%), there was concordance (i.e., a unidirectional increase or decrease) in the BrdU incorporation results after HDR‐BT between Huh7 and HepG2. Specifically, 11 of 12 responders (91.7%) as well as nine out of 11 nonresponders (81.8%) demonstrated a unidirectional increase or decrease in BrdU incorporation between the two cell lines.

### 3.3 Effects of serum from responders and nonresponders on cyclin E expression

**Fig. 4:**
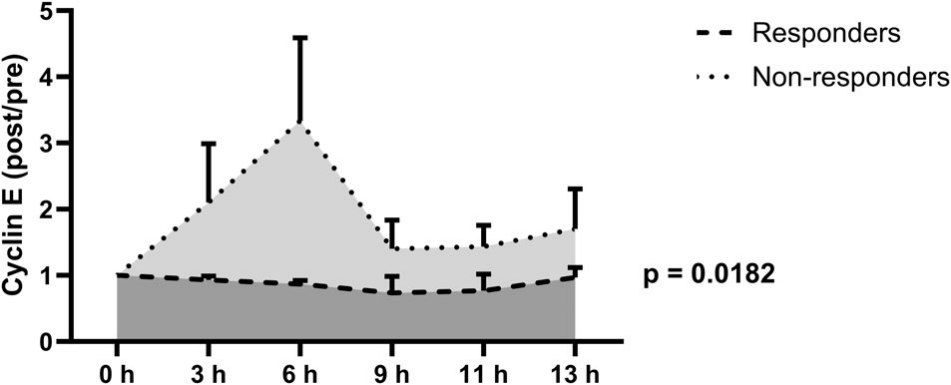
Cyclin E expression in synchronized HepG2 cells exposed to serum collected from responders and nonresponders. Cells were synchronized by nutrient starvation for 24 h and consequently incubated with serum before and after high dose rate brachytherapy (HDR‐BT) from a representative subgroup of responders (*n* = 3) and nonresponders (*n* = 3). The experiment was performed once. Immunodetection of cyclin E was performed at different time points, rendering a ratio of Cyclin E expression after and before HDR‐BT (Cyclin E expression post/pre) for each patient. Elevated Cyclin E expression ratios were observed after cell cycle induction with serum of nonresponders compared to responders. Interaction of response and time showed a significant influence on Cyclin E expression ratios in a spline linear mixed model (*P* = 0.0182). Peak Cyclin E expression ratio was noticed at 6 h after cell cycle induction with serum in nonresponders. A spline linear mixed model was used to compare responders and nonresponders. Results are reported as mean and standard deviation.

The effect of serum collected from responders and nonresponders was evaluated via the expression level of Cyclin E after cell cycle induction in order to validate the findings obtained with the BrdU incorporation assay through a second outcome parameter. In HepG2 cells, FCSdeprivation showed a significant reduction of BrdU incorporation (median FCS‐supplemented: 0.56 [0.51–0.59], FCS‐deprived: 0.06 [0.05–0.07], *P* = 0.0079, Fig. S3). By contrast, in Huh7 cells, FCSdeprivation did not lead to a reduction but rather to a slight, non‐significant increase of BrdU incorporation (FCS‐supplemented: 0.54 [0.41–0.58], FCS‐deprived: 0.66 [0.57–0.72], *P* = 0.1508). HepG2 cells incubated with the serum of nonresponders showed increased Cyclin E expression ratios compared to the serum of responders when applying a two‐knot spline linear mixed model respecting response, time, and interaction of time and response (Fig. 4, interaction of time and response: *P* = 0.0182). Moreover, the model showed a time‐dependent change of Cyclin E expression ratios to 6 h (*P* = 0.0132) and a statistical trend to difference between 6 and 9 h (*P* = 0.0777) in nonresponders while expression ratios in responders remained close to baseline at all time points.

### 3.4 BrdU incorporation predicts opposite responses and indicates altered TTSP in HCC patients undergoing HDR‐BT

**Fig. 5:**
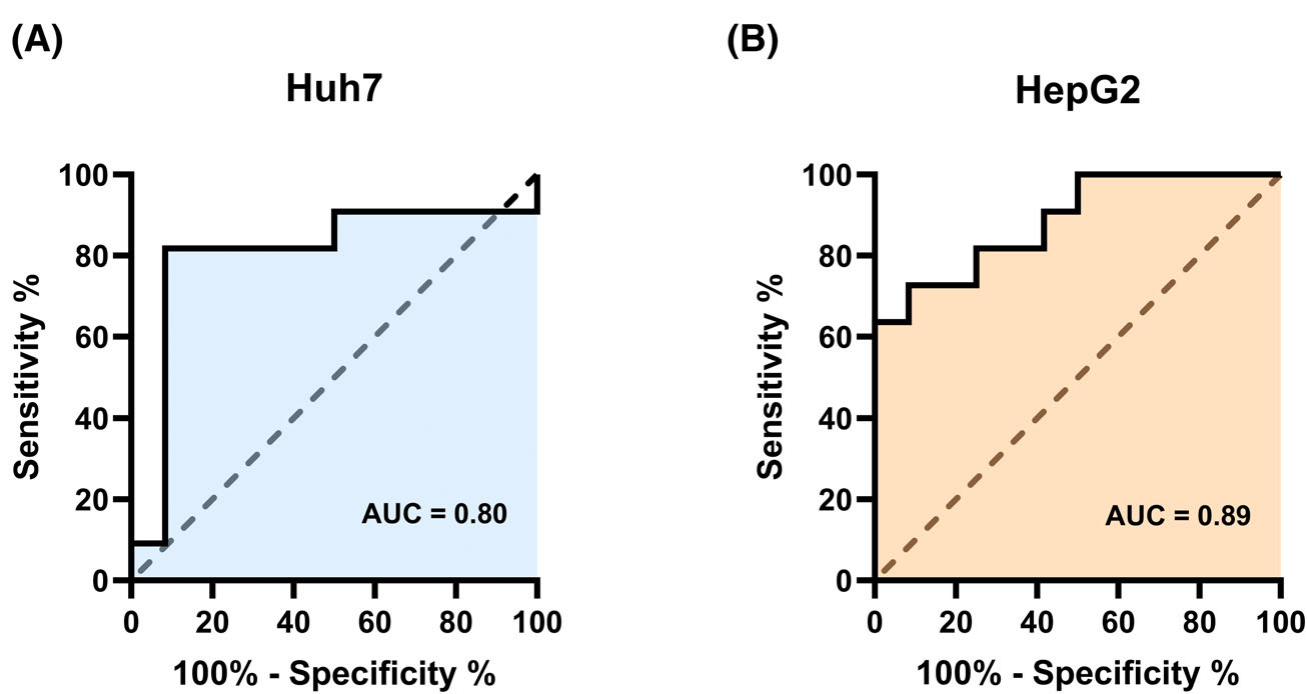
ROC curves of BrdU incorporation to predict therapy response. In Huh7 (A), the Receiver Operating Characteristic (ROC) Curve illustrates the performance of the classification model in distinguishing between responders and nonresponders. ROC operating points were approximated to the maximum sum of sensitivity and specificity. Corresponding metrics are illustrated. The area under the curve (AUC) is 0.80 for Huh7 (A) and 0.89 for HepG2 (B). For each cell line, two independent experiments were performed to assess BrdU incorporation.

Relative to BrdU incorporation ratios, Receiver Operating Characteristics yielded an AUC of 0.80 for Huh7 and 0.89 for HepG2 to distinguish between responders and nonresponders, respectively (Fig. 5A,B). For Huh7, the definition of a threshold at 1.04 as a BrdU incorporation ratio cut‐off (threshold optimization, Youden statistics) achieved a sensitivity/specificity of 0.92/0.82, two false positives with a positive predictive value of 0.85, as well as one false negative with a negative predictive value of 0.90, rendering a Youden's *J* of 0.74.

For HepG2, the definition of a threshold at 0.91 for a BrdU incorporation ratio cut‐off (threshold optimization, Youden statistics) achieved a sensitivity/specificity of 0.92/0.73, 3 false positives with a positive predictive value of 0.79, as well as 1 false negative with a negative predictive value of 0.89, rendering a Youden's *J* of 0.64.

TTSP of patients stratified by BrdU incorporation ratios < 0.9 and > 1.1 differed significantly between the two groups for both cell lines.

**Fig. 6:**
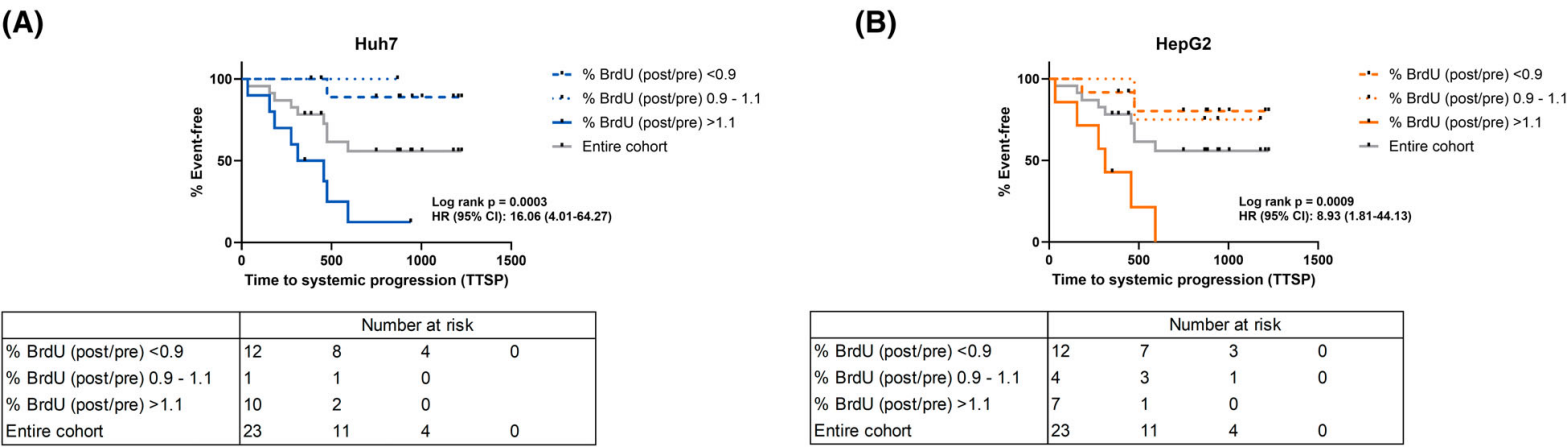
TTSP in accordance with BrdU incorporation. Kaplan–Meier curves display time to systemic progression (TTSP) for patients with BrdU incorporation ratios of < 0.9, from 1.1 to 0.99, > 1.1 and for the entire patient cohort. Patients with a BrdU incorporation ratio of > 1.1 demonstrated significantly shorter TTSP than patients with a BrdU incorporation ratio of < 0.9 (*P* = 0.0003, A; *P* = 0.0009, B; for Huh7 and HepG2, respectively). Log‐rank and Hazard Ratio statistics are depicted for the comparison of the groups with BrdU incorporation ratios of > 1.1 and < 0.9. For each cell line, two independent experiments were performed to assess BrdU incorporation.

For Huh7, patients with a BrdU incorporation ratio > 1.1 presented with a median TTSP of 385 days. In patients with a BrdU incorporation ratio < 0.9, it could not be calculated as less than 50% of patients showed progression during study follow‐up (Fig. 6A, HR 16.06, 95% CI 4.01–64.27, *P* = 0.0003).

For HepG2, patients with a BrdU incorporation ratio > 1.1 presented with a median TTSP of 312 days. In patients with a BrdU incorporation ratio < 0.9, within the study follow‐up could not be calculated as less than 50% of patients showed progression during study follow‐up (Fig. 6B, HR 8.93, 95% CI 1.81–44.13, *P* = 0.0009).

Combined analyses of the group with BrdU incorporation in Huh7 and/or HepG2 > 0.9 revealed a median TTSP of 312 days. In patients whose serum produced a BrdU incorporation ratio < 0.9 in both cell lines, the median TTSP could not be calculated as less than 50% of patients showed progression during study follow‐up (Fig. S4, *P* = 0.0003).

### 3.5 BrdU incorporation is associated with PTN and CRTAM levels in patient plasma

Correlation of the BrdU incorporation ratios with all tested plasma proteins showed the strongest association for the proteins Pleiotrophin (PTN) and Cytotoxic and regulatory T Cell Molecule (CRTAM) among a 92‐protein panel of immune‐oncology related cytokines with the two investigated cell lines rendering a mean Spearman *r* of > 0.5. Post/Pre protein ratios of patients showed a stronger correlation when patients were stratified according to their BrdU incorporation ratio compared to when stratified based upon clinical response (Fig. S5). Plasma PTN ratios in patients stratified according to the patients' BrdU incorporation ratio rendered a more notable statistical difference (Huh7: *P* = 0.0026, HepG2: *P* = 0.0034) compared to when stratified according to the patients' response (*P* = 0.0255). Of note, plasma CRTAM ratios did not achieve significant difference when stratified according to response (*P* = 0.4959) but rendered significant differences when stratified according to BrdU incorporation ratio in HepG2 (*P* = 0.0056) with a trend to significance in Huh7 (*P* = 0.0764).

## 4 Discussion

Our study shows that altered DNA synthesis by the means of BrdU incorporation in hepatoma cell lines following exposure to patient serum can serve as a potential surrogate biomarker for response to local tumor ablation. Compared to baseline, serum obtained after HDR‐BT from responders renders a decrease in BrdU incorporation, whereas nonresponders demonstrate increased BrdU incorporation. Beyond this dichotomic clinical association, these observations enable the successful stratification of patients with regard to their TTSP. Moreover, these results confirm the hypothesis that serum from patients with poor outcomes increases DNA synthesis, whereas serum from patients with beneficial outcomes decreases DNA synthesis.

To the best of our knowledge, our investigation is the first study indicating a proliferative, potentially oncogenic effect of patient serum after local tumor ablation. Interestingly, the robustness of these results is underlined by a high level of concordance between both studied cell lines, as the majority of patients showed similar BrdU incorporation ratios after serum incubation between the two cell lines. Our study provides evidence in several phases of the cell cycle. Differences in responders and nonresponders could not only be shown by the level of DNA synthesis but also by the amount of Cyclin E expression, with the latter indicating the transition from G1 to S‐phase in exposed hepatoma cells.

The herein‐described experimental setup of incubating target cells with patient serum has previously been applied similarly for various tumor entities [10]. Specifically, in the context of liver diseases, this approach was carried out for research questions regarding chronic inflammation in the context of Hepatitis B, suggesting insulin‐like growth factor facilitating HCC growth [11] or oncologic surgery to demonstrate the influence of Hepatocyte growth factor on HCC cell growth *in vitro* [12]. By applying a similar experimental setup, our study advances the current understanding of local tumor ablation and its associated systemic effects. We now show a direct effect mediated by patient serum after HDR‐BT, indicating the presence of multiple pro‐tumorigenic factors with cell cycle activity. Independent of individual proteins, the entirety of the molecules contained in the patient serum could serve as an additional valuable tool for assessing the growth potential of tumors after ablation.

So far, mainly individual factors or signaling pathways altered after local tumor ablation have been investigated. In thermal ablation, multiple molecules might be needed to detect post‐ablation tumor growth patterns [1]. The previous analysis of the herein investigated cohort revealed elevated PDGF‐B and EGF associated with poor outcome [6]. Other analyses confirm proteins of tumor growth and angiogenesis, such as VEGF or TGF‐β [13, 14]. Interestingly, our results show a strong correlation of PTN and CRTAM with BrdU incorporation for both investigated cell lines, suggesting that these two proteins may very well contribute to cell proliferation after HDR‐BT. An association between elevated PTN and increased BrdU incorporation is in concert with the literature, as it is described as a pro‐tumorigenic protein in HCC and other tumor entities [15, 16, 17]. Our finding of the association of increased CRTAM with increased BrdU incorporation remains challenging to interpret, as current literature on CRTAM in the context of HCC is scarce. In other tumor entities, however, CRTAM has been reported to be associated with antitumor immune response on the transcriptional level [18, 19]. Besides these proteins, the literature suggests that other factors might contribute to the treatment response of HCC after radiation. Radiation treatment was shown to upregulate PD‐L1 in human and murine HCC cells via the cGAS‐STING signaling pathway [20] and PD‐L1‐related immune escape is mediated through the HGF/c‐Met axis [21]. Interestingly, local ablation can increase HCC proliferation via HGF/c‐Met signaling [22, 23], which may reflect a potential mechanistic link between the impact of known tumorigenic factors on HCC growth *in vitro* and poor clinical outcome, mediated through patient serum after local ablation.

The here presented association between BrdU incorporation ratio and increased plasma PTN and CRTAM after HDR‐BT emphasizes the need for further investigation, especially determination of by which cells these factors are secreted and in which tissue compartments they can be detected best. Furthermore, epigenetic alterations and overexpression of genes can negatively influence oncologic outcome following local tumor ablation [3, 24, 25]. Besides pro‐tumorigenic factors, senescence‐associated pathways initiated by stromal cells may influence HCC growth by creating an immunosuppressive tumor microenvironment [26]. In contrast to the aforementioned described oncogenic factors following local tumor treatment, the herein‐described translational setup not only reflects isolated singular factors but rather demonstrates the influence of potentially multiple molecular pathways contributing to tumor growth.

In clinical practice, the findings of this study could help to distinguish between HCC patients who have the potential for favorable response from those not responding to HDR‐BT. By offering predictive capabilities and suggesting patient outcomes, the described experimental set‐up could provide a beneficial predictive biomarker of treatment success already shortly after the procedure. Indeed, the reported method of patient stratification according to the BrdU incorporation using a combination of both cell lines would provide a robust tool to predict shorter or extended TTSP. Consequently, differentiation between patients who are likely to benefit from local tumor ablation and those at higher risk of early recurrence could be carried out. Identification of such patients at risk would, for instance, indicate the need for closer follow‐up monitoring and adjuvant therapy. With further validation in larger cohorts, this enhanced clinical decision‐making could lead to improved patient outcomes and better survival after HDR‐BT.

This study has several limitations. First, the described findings are limited to the observation of serum incubation effects after HDR‐BT of HCC, while other techniques of local tumor ablation and other tumor entities remain as yet unexplored, which calls for further research. We speculate that the study of other hepatic malignancies with more aggressive growth patterns, such as colorectal carcinoma, might lead to more pronounced proliferative effects [27, 28]. Second, additional outcome parameters beyond BrdU incorporation were not performed for all 23 patients. Nevertheless, Cyclin E expression was measured for representative patients and confirmed the validity of the results obtained by serum incubation of two different cell lines. Lastly, and most importantly, no specific molecular pathway has been elucidated in this study. Nevertheless, we emphasize that this approach could provide an additional tool to assess post‐therapeutic proliferation capability, as it potentially reflects multiple growth pathways. However, a comprehensive understanding of the underlying mechanisms of the reported radiation‐induced findings remains unknown and requires further elucidation. Accordingly, further investigation, for example, by specific inhibition of key oncogenic molecules and their respective impact on tumor growth, is warranted. Such identification of responsible targets could lay the groundwork for targeted pharmaceutical therapy by inhibition of pro‐tumorigenic factors.

## 5 Conclusions

This study demonstrates the alteration of proliferation parameters in two different hepatoma cell lines after exposure to patient serum before and after local tumor ablation and provides initial evidence for its predictive capabilities regarding clinical outcome.

## Conflict of interest

The following authors received funding or consulting fees not related to this research. LS received funding from Deutsche Forschungsgesellschaft. SNG received funding from Israel Science Foundation and consulting fees from CAPS medical, SNIPE medical, Cambridge International. EÖ received funding from the Society of Interventional Oncology. SC received funding from Elekta, Brainlab, Viewray and consulting fees from Elekta, Brainlab, Viewray. NBK received consulting fees from Falk Foundation, Astra Zeneca. MSe received funding from Astra Zeneca, Boston Scientific (institutional) and consulting fees from Cook medical, SIRTEX, Astra Zeneca, Roche, Siemens, Bayer.

## Author contributions

All authors have made substantial contributions and satisfy the criteria for authorship: conception and design; analysis and interpretation of the data; drafting of the article; critical revision of the article for important intellectual content; final approval of the article. All agree to be accountable for all aspects of the work in ensuring that questions related to the accuracy or integrity of any part of the work are appropriately investigated and resolved. We confirm that there are no other persons who satisfied the criteria for authorship but are not listed. We understand that the corresponding author is the sole contact for the editorial process. The work reported in the paper has been performed by the authors, unless clearly specified in the text. Conceptualization: LS, SNG, JR, MSt, MA‐F. Data curation: LS, JNS, MNG, TB, JR, MSt, MA‐F. Formal analysis: LS, JNS, SNG, MNG, PFL, EÖ, TB, NBK, JR, MSt, MA‐F. Investigation: LS, JNS, PMK, MNG, PFL, EÖ, SC, NBK, MW, MSe, JR, MSt, MA‐F. Methodology: LS, JNS, SNG, PMK, EÖ, TB, JR, MSt, MA‐F. Supervision: SNG, PMK, SC, MW, MSe, JR, MSt, MA‐F. Drafting of the manuscript: LS, JNS, SNG, MNG, PFL, MW, MSe, JR, MSt, MA‐F. Critical revision of the manuscript for important intellectual content: All.

## Supporting information


**Fig. S1.** BrdU incorporation after serum incubation in responders and nonresponders.
**Fig. S2.** Comparison of BrdU incorporation levels between responders and nonresponders.
**Fig. S3.** FCS‐deprivation in Huh7 and HepG2 cells.
**Fig. S4.** Time to systemic progression (TTSP) in accordance with BrdU incorporation for combined analysis of Huh7 and HepG2.
**Fig. S5.** Heatmap illustrating the intensity of BrdU incorporation and plasma protein levels per patient.
**Table S1.** Clinical and technical characteristics of the observed 23 HCC patients undergoing HDR‐BT.
**Table S2.** Laboratory baseline parameters of the observed 23 HCC patients undergoing HDR‐BT.
**Table S3.** (A) Absolute BrdU incorporation after serum incubation of Huh7. (B) Absolute BrdU incorporation after serum incubation of HepG2.

## Data Availability

The data that support the findings of this study are openly available in ‘Dryad’ at https://doi.org/10.5061/dryad.9p8cz8wt0.
