# Supplementary material for: In vitro properties of patient serum predict clinical outcome after high dose rate brachytherapy of hepatocellular carcinoma
Source: Mol Oncol. 2025 Sep 12;20(2):480–92. doi: 10.1002/1878-0261.70122 (PMC12936419; doi:10.1002/1878-0261.70122)
Supplement: Supplementary file 1 — Fig. S1. BrdU incorporation after serum incubation in responders and nonresponders. Fig. S2. Comparison of BrdU incorporation levels between responders and nonresponders. Fig. S3. FCS‐deprivation in Huh7 and HepG2 cells. Fig. S4. Time to systemic progression (TTSP) in accordance with BrdU incorporation for combined analysis of Huh7 and HepG2. Fig. S5. Heatmap illustrating the intensity of BrdU incorporation and plasma protein levels per patient. Table S1. Clinical and technical characteristics of the observed 23 HCC patients undergoing HDR‐BT. Table S2. Laboratory baseline parameters of the observed 23 HCC patients undergoing HDR‐BT. Table S3. (A) Absolute BrdU incorporation after serum incubation of Huh7. (B) Absolute BrdU incorporation after serum incubation of HepG2. [file MOL2-20-480-s001.zip › Supporting information.docx]

**Supplementary Figures**

**Supplementary Figure 1. BrdU incorporation after serum incubation in responders and non-responders.** Stacked bar plots show significantly different fractions of responders and non-responders with all increased (>1.0; hatched) or decreased (<1.0; unfilled) BrdU incorporation for Huh7 (A) and HepG2 (B). For each cell line, two independent experiments were performed to assess BrdU incorporation.

**Supplementary Figure 2. Comparison of BrdU incorporation levels between responders and non-responders.** Box plots illustrating BrdU incorporation ratios (post/pre therapy) in Huh7 (A, blue) and HepG2 cells (B, orange). In Huh7, responders showed significantly lower levels compared to non-responders (p=0.0158). Likewise, in HepG2, responders showed significantly lower levels compared to non-responders (p=0.0004). For each cell line, two independent experiments were performed to assess BrdU incorporation.

**Supplementary Figure 3. FCS-deprivation in Huh7 and HepG2 cells.** Box plots illustrating the BrdU incorporation after an incubation period of 24 h with either FCS-supplemented or FCS-deprived medium. After incubation with FCS-deprived medium Huh7 cells show a slight, non-significant increase of BrdU incorporation compared to incubation with FCS-supplemented medium (p=0.1508). By contrast, HepG2 cells demonstrate a significant decrease after incubation with FCS-deprived compared to FCS-supplemented medium (p=0.0079).

**Supplementary Figure 4. Time to systemic progression (TTSP) in accordance with BrdU incorporation for combined analysis of Huh7 and HepG2.** All patients with a BrdU incorporation ratio <0.9 in both cell lines, compared to all other patients not fulfilling this criterion, showed significantly longer TTSP (p=0.0003). For each cell line, two independent experiments were performed to assess BrdU incorporation.

**Supplementary Figure 5. Heatmap illustrating the intensity of BrdU incorporation and plasma protein levels per patient.** Post/pre HDR-BT ratios of BrdU incorporation in Huh7 and HepG2, as well as post/pre ratios of the plasma proteins PTN and CRTAM are shown per individual patient. All values were log2-transformed and z-score normalized for appropriate visualization. Positive cells (red) represent a post/pre ratio >1, indicating an increase after HDR-BT, whereas negative cells (blue) represent a post/pre ratio <1, indicating a decrease after HDR-BT.

**Supplementary Tables**

|  | Overall/median (%/range IQR) | Responders number/median (%/range IQR) | Non-Responders number/median (%/range IQR) | p-value |
| --- | --- | --- | --- | --- |
| **Basic characteristics** | |  |  |  |
| Female | 4 (17.4) | 2 (16.7) | 2 (18.2) | 1 |
| Male | 19 (82.6) | 10 (83.3) | 9 (81.8) |  |
| Age | 69 (59-80 (21)) | 69 (60-80 (20)) | 72 (56-78 (22)) | 0.8679 |
|  |  |  |  |  |
| **Tumor etiology** |  |  |  |  |
| Hepatitis B | 3 (13.0) | 1 (8.3) | 2 (18.2) | 0.5901 |
| Hepatitis C | 2 (8.7) | 2 (16.7) | 1 (9.1) | 1 |
| Alcoholic | 5 (21.7) | 3 (25) | 2 (18.2) | 0.6668 |
| NASH | 3 (13.0) | 1 (8.3) | 2 (18.2) | 0.5901 |
| Unknown | 6 (26.1) | 3 (25.0) | 3 (27.3) | 1 |
| Multiple | 3 (13.0) | 2 (16.7) | 1 (9.1) | 1 |
|  |  |  |  |  |
| **Tumor characteristics** | |  |  |  |
| Child-Pugh A | 18 (78.3) | 10 (83.3) | 8 (72.7) | 0.6404 |
| Child-Pugh B | 5 (21.7) | 2 (16.7) | 3 (27.3) |  |
| AFP (ng/ml) | 5.3 (3.55-9.55 (6)) | 5.3 (3.8-7.8 (4)) | 5.7 (3.1-26.7 (23.6)) | 0.5759 |
| AFP >20 ng/ml | 4 (17.4) | 1 (8.3) | 3 (27.3) | n.a. |
| TTSP | 475 (369-912 (543)) | 912 (838-1048 (210)) | 351 (229-466 (237)) | 0.0001 |
|  |  |  |  |  |
| **Technical parameters** | |  |  |  |
| Lesion diameter sum (cm) | 3 (2.45-5.45 (3)) | 2.9 (2.5-3.28 (0.78)) | 4 (2.55-6.9 (4.35)) | 0.2668 |
| Lesion diameter max (cm) | 2.5 (2.15-3.35 (1.2)) | 2.65 (2.45-3.05 (0.6)) | 2.4 (1.7-6.9 (5.2)) | 0.4756 |
| Lesion number treated: 1 | 15 (65.2) | 10 (83.3) | 5 (45.5) | 0.0894 |
| Lesion number treated: 2 | 8 (34.7) | 2 (16.7) | 6 (54.5) |  |

**Supplementary Table 1. Clinical and technical characteristics of the observed 23 HCC patients undergoing HDR-BT.**

| *Variable* | *Response* | *n* | *Mean* | *Standard deviation* | *Minimum* | *25th Percentile* | *50th percentile (Median)* | *75th percentile* | *Maximum* | *p-value* |
| --- | --- | --- | --- | --- | --- | --- | --- | --- | --- | --- |
| AFP | no | 11 | 3290.15 | 10767.56 | 2.00 | 2.60 | 5.70 | 42.90 | 35754.00 |  |
|  | yes | 12 | 6.86 | 5.62 | 1.60 | 3.75 | 5.25 | 8.05 | 21.50 | 0.579 |
| ALAT | no | 11 | 47.45 | 33.58 | 16.00 | 22.00 | 33.00 | 81.00 | 112.00 |  |
|  | yes | 12 | 34.25 | 15.40 | 12.00 | 23.50 | 31.50 | 42.00 | 67.00 | 0.558 |
| ASAT | no | 11 | 68.45 | 34.88 | 32.00 | 37.00 | 73.00 | 92.00 | 134.00 |  |
|  | yes | 12 | 54.42 | 19.05 | 26.00 | 36.50 | 53.00 | 71.00 | 83.00 | 0.372 |
| Albumin | no | 11 | 3.65 | 0.57 | 2.60 | 3.30 | 3.60 | 4.20 | 4.50 |  |
|  | yes | 12 | 3.88 | 0.48 | 2.80 | 3.75 | 4.10 | 4.20 | 4.30 | 0.306 |
| Cholinesterase | no | 9 | 4.89 | 2.17 | 2.17 | 3.70 | 4.91 | 5.71 | 9.30 |  |
|  | yes | 11 | 4.76 | 1.38 | 2.52 | 4.20 | 4.39 | 5.80 | 7.65 | 0.970 |
| GLDH | no | 9 | 16.67 | 25.73 | 3.50 | 3.80 | 7.90 | 9.50 | 83.30 |  |
|  | yes | 9 | 9.40 | 8.58 | 2.90 | 3.70 | 5.70 | 9.20 | 27.40 | 0.536 |
| Gamma-GT | no | 11 | 248.73 | 328.29 | 36.00 | 64.00 | 139.00 | 275.00 | 1145.00 |  |
|  | yes | 12 | 184.42 | 141.95 | 32.00 | 71.50 | 169.00 | 214.50 | 492.00 | 0.853 |
| INR | no | 11 | 1.13 | 0.17 | 0.90 | 1.00 | 1.10 | 1.20 | 1.50 |  |
|  | yes | 12 | 1.12 | 0.15 | 0.90 | 1.05 | 1.10 | 1.15 | 1.50 | 0.700 |
| Creatinine | no | 11 | 0.93 | 0.29 | 0.70 | 0.70 | 0.90 | 1.10 | 1.60 |  |
|  | yes | 12 | 1.02 | 0.34 | 0.60 | 0.90 | 0.95 | 1.05 | 2.00 | 0.416 |
| Lymphocytes | no | 9 | 1.66 | 0.75 | 0.64 | 0.96 | 1.56 | 2.37 | 2.63 |  |
|  | yes | 11 | 1.19 | 0.45 | 0.45 | 0.83 | 1.19 | 1.62 | 1.92 | 0.196 |
| N/L ratio | no | 9 | 2.47 | 1.18 | 1.07 | 1.46 | 2.46 | 2.82 | 4.84 |  |
|  | yes | 11 | 3.69 | 1.31 | 2.11 | 2.77 | 3.32 | 4.56 | 6.04 | 0.068 |
| Sodium | no | 11 | 139.45 | 3.24 | 135.00 | 136.00 | 139.00 | 142.00 | 145.00 |  |
|  | yes | 12 | 138.92 | 2.54 | 134.00 | 137.50 | 138.50 | 141.00 | 143.00 | 0.598 |
| Neutrophils | no | 9 | 3.54 | 1.41 | 1.79 | 2.81 | 3.27 | 3.45 | 6.77 |  |
|  | yes | 11 | 4.04 | 1.21 | 2.43 | 2.90 | 3.95 | 4.99 | 6.10 | 0.362 |
| Prothrombin time | no | 10 | 0.85 | 0.19 | 0.51 | 0.72 | 0.83 | 0.95 | 1.16 |  |

**Supplementary Table 2. Laboratory baseline parameters of the observed 23 HCC patients undergoing HDR-BT.** Note that the neutrophil-to-lymphocyte ratio (N/L ratio) was performed with n=20 patients (for patients with ID 3, 15, 19 this data has not been obtained).

| Patient ID | Response | Experiment 1 | | | Experiment 2 | | | Combined | | |
| --- | --- | --- | --- | --- | --- | --- | --- | --- | --- | --- |
|  |  | Pre therapy | Post therapy | Ratio Post/Pre | Pre therapy | Post therapy | Ratio Post/Pre | Pre therapy | Post therapy | Ratio Post/Pre |
| 1 | Responder | 1.86 | 0.68 | 0.37 | 9.78 | 8.30 | 0.85 | 5.82 | 4.49 | 0.61 |
| 2 | Responder | 3.06 | 2.18 | 0.71 | 10.34 | 7.06 | 0.68 | 6.70 | 4.62 | 0.70 |
| 3 | Responder | 5.22 | 2.20 | 0.42 | 9.18 | 5.66 | 0.62 | 7.20 | 3.93 | 0.52 |
| 4 | Responder | 6.82 | 3.84 | 0.56 | 10.32 | 6.02 | 0.58 | 8.57 | 4.93 | 0.57 |
| 5 | Responder | 0.46 | 5.90 | 12.83 | 4.88 | 13.02 | 2.67 | 2.67 | 9.46 | 7.75 |
| 6 | Responder | 10.96 | 7.10 | 0.65 | 13.48 | 4.66 | 0.35 | 12.22 | 5.88 | 0.50 |
| 7 | Responder | 8.84 | 3.22 | 0.36 | 5.72 | 4.78 | 0.84 | 7.28 | 4.00 | 0.60 |
| 8 | Responder | 10.04 | 3.10 | 0.31 | 4.78 | 3.78 | 0.79 | 7.41 | 3.44 | 0.55 |
| 9 | Responder | 5.66 | 0.70 | 0.12 | 10.34 | 4.52 | 0.44 | 8.00 | 2.61 | 0.28 |
| 10 | Responder | 7.68 | 1.60 | 0.21 | 15.88 | 6.40 | 0.40 | 11.78 | 4.00 | 0.31 |
| 11 | Responder | 3.54 | 2.96 | 0.84 | 12.24 | 8.24 | 0.67 | 7.89 | 5.60 | 0.75 |
| 12 | Responder | 3.12 | 1.34 | 0.43 | 18.42 | 11.10 | 0.60 | 10.77 | 6.22 | 0.52 |
| 13 | Non-responder | 0.40 | 4.10 | 10.25 | 4.34 | 7.34 | 1.69 | 2.37 | 5.72 | 5.97 |
| 14 | Non-responder | 1.68 | 3.96 | 2.36 | 5.16 | 13.66 | 2.65 | 3.42 | 8.81 | 2.50 |
| 15 | Non-responder | 1.34 | 2.00 | 1.49 | 2.20 | 6.42 | 2.92 | 1.77 | 4.21 | 2.21 |
| 16 | Non-responder | 7.38 | 5.22 | 0.71 | 9.28 | 6.86 | 0.74 | 8.33 | 6.04 | 0.72 |
| 17 | Non-responder | 2.42 | 5.50 | 2.27 | 5.14 | 12.12 | 2.36 | 3.78 | 8.81 | 2.32 |
| 18 | Non-responder | 7.96 | 2.28 | 0.29 | 15.06 | 6.80 | 0.45 | 11.51 | 4.54 | 0.37 |
| 19 | Non-responder | 7.18 | 8.36 | 1.16 | 8.46 | 10.42 | 1.23 | 7.82 | 9.39 | 1.20 |
| 20 | Non-responder | 11.68 | 0.94 | 0.08 | 3.28 | 6.98 | 2.13 | 7.48 | 3.96 | 1.10 |
| 21 | Non-responder | 2.92 | 6.58 | 2.25 | 10.84 | 11.22 | 1.04 | 6.88 | 8.90 | 1.64 |
| 22 | Non-responder | 2.86 | 9.04 | 3.16 | 6.26 | 9.42 | 1.50 | 4.56 | 9.23 | 2.33 |
| 23 | Non-responder | 1.08 | 12.08 | 11.19 | 4.82 | 19.44 | 4.03 | 2.95 | 15.76 | 7.61 |

**Supplementary Table 3A. Absolute BrdU incorporation after serum incubation of Huh7.** Indicated are the absolute values of BrdU incorporation before (pre-therapy) and after HDR-BT (post-therapy), as well as the ratio created from both time points (Ratio post/pre) for each patient.

| Patient ID | Response | Experiment 1 | | | Experiment 2 | | | Combined | | |
| --- | --- | --- | --- | --- | --- | --- | --- | --- | --- | --- |
|  |  | Pre therapy | Post therapy | Ratio Post/Pre | Pre therapy | Post therapy | Ratio Post/Pre | Pre therapy | Post therapy | Ratio Post/Pre |
| 1 | Responder | 8.28 | 5.74 | 0.69 | 2.70 | 0.80 | 0.30 | 5.49 | 3.27 | 0.49 |
| 2 | Responder | 8.54 | 9.78 | 1.15 | 3.42 | 2.28 | 0.67 | 5.98 | 6.03 | 0.91 |
| 3 | Responder | 3.18 | 1.74 | 0.55 | 0.34 | 0.16 | 0.47 | 1.76 | 0.95 | 0.51 |
| 4 | Responder | 5.42 | 2.62 | 0.48 | 2.08 | 1.58 | 0.76 | 3.75 | 2.10 | 0.62 |
| 5 | Responder | 4.48 | 8.18 | 1.83 | 0.50 | 0.12 | 0.24 | 2.49 | 4.15 | 1.03 |
| 6 | Responder | 8.76 | 7.50 | 0.86 | 2.98 | 0.24 | 0.08 | 5.87 | 3.87 | 0.47 |
| 7 | Responder | 5.52 | 3.70 | 0.67 | 0.58 | 0.18 | 0.31 | 3.05 | 1.94 | 0.49 |
| 8 | Responder | 5.48 | 3.74 | 0.68 | 3.04 | 0.64 | 0.21 | 4.26 | 2.19 | 0.45 |
| 9 | Responder | 2.96 | 2.56 | 0.86 | 0.40 | 0.40 | 1.00 | 1.68 | 1.48 | 0.93 |
| 10 | Responder | 6.22 | 7.26 | 1.17 | 0.38 | 0.10 | 0.26 | 3.30 | 3.68 | 0.72 |
| 11 | Responder | 7.76 | 8.78 | 1.13 | 0.96 | 0.54 | 0.56 | 4.36 | 4.66 | 0.85 |
| 12 | Responder | 11.50 | 3.24 | 0.28 | 1.48 | 0.26 | 0.18 | 6.49 | 1.75 | 0.23 |
| 13 | Non-responder | 8.98 | 7.12 | 0.79 | 0.10 | 0.90 | 9.28 | 4.54 | 4.01 | 5.04 |
| 14 | Non-responder | 2.20 | 5.26 | 2.39 | 0.44 | 0.52 | 1.18 | 1.32 | 2.89 | 1.79 |
| 15 | Non-responder | 3.34 | 6.76 | 2.02 | 1.64 | 1.64 | 1.00 | 2.49 | 4.20 | 1.51 |
| 16 | Non-responder | 7.96 | 6.62 | 0.83 | 0.10 | 0.16 | 1.60 | 4.03 | 3.39 | 1.22 |
| 17 | Non-responder | 9.44 | 5.32 | 0.56 | 1.96 | 0.86 | 0.44 | 5.70 | 3.09 | 0.50 |
| 18 | Non-responder | 4.02 | 3.98 | 0.99 | 3.94 | 2.64 | 0.67 | 3.98 | 3.31 | 0.83 |
| 19 | Non-responder | 9.56 | 8.64 | 0.90 | 1.26 | 0.64 | 0.51 | 5.41 | 4.64 | 0.71 |
| 20 | Non-responder | 6.36 | 2.14 | 0.34 | 0.30 | 2.62 | 8.73 | 3.33 | 2.38 | 4.53 |
| 21 | Non-responder | 2.38 | 4.04 | 1.70 | 0.22 | 1.52 | 6.91 | 1.30 | 2.78 | 4.30 |
| 22 | Non-responder | 1.28 | 1.68 | 1.31 | 0.28 | 0.82 | 2.93 | 0.78 | 1.25 | 2.12 |
| 23 | Non-responder | 2.62 | 5.34 | 2.04 | 0.20 | 1.00 | 5.00 | 1.41 | 3.17 | 3.52 |

**Supplementary Table 3B. Absolute BrdU incorporation after serum incubation of HepG2.** Indicated are the absolute values of BrdU incorporation before (pre-therapy) and after HDR-BT (post-therapy), as well as the ratio created from both time points (Ratio post/pre) for each patient.
