# Supplementary figures and images for: In vitro properties of patient serum predict clinical outcome after high dose rate brachytherapy of hepatocellular carcinoma
Source: Mol Oncol. 2025 Sep 12;20(2):480–92. doi: 10.1002/1878-0261.70122 (PMC12936419; doi:10.1002/1878-0261.70122)

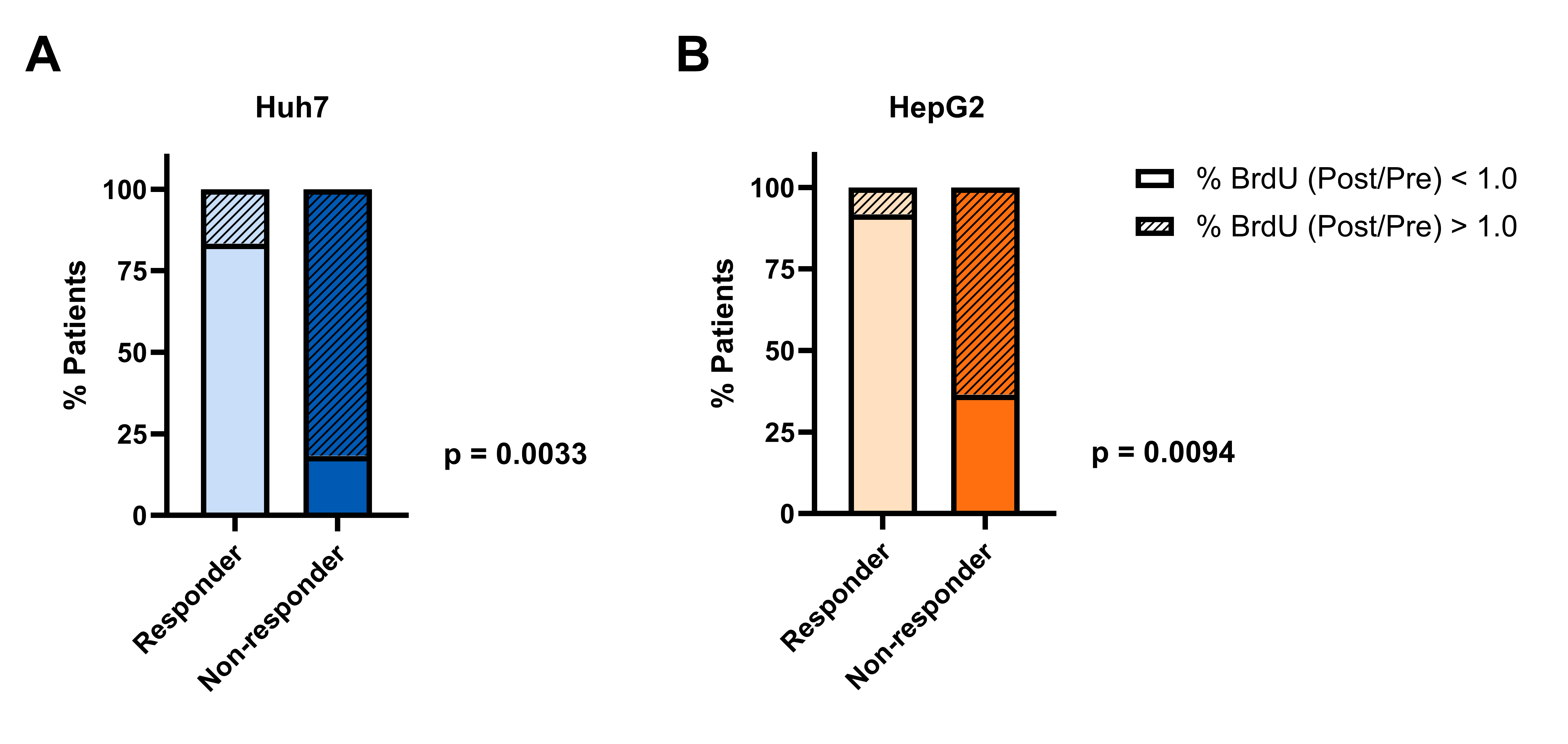

Supplement: Supplementary file 1 — Fig. S1. BrdU incorporation after serum incubation in responders and nonresponders. Fig. S2. Comparison of BrdU incorporation levels between responders and nonresponders. Fig. S3. FCS‐deprivation in Huh7 and HepG2 cells. Fig. S4. Time to systemic progression (TTSP) in accordance with BrdU incorporation for combined analysis of Huh7 and HepG2. Fig. S5. Heatmap illustrating the intensity of BrdU incorporation and plasma protein levels per patient. Table S1. Clinical and technical characteristics of the observed 23 HCC patients undergoing HDR‐BT. Table S2. Laboratory baseline parameters of the observed 23 HCC patients undergoing HDR‐BT. Table S3. (A) Absolute BrdU incorporation after serum incubation of Huh7. (B) Absolute BrdU incorporation after serum incubation of HepG2. [file MOL2-20-480-s001.zip › MOL2_70122_f1_SUPPL_FIG1.TIF.tif]

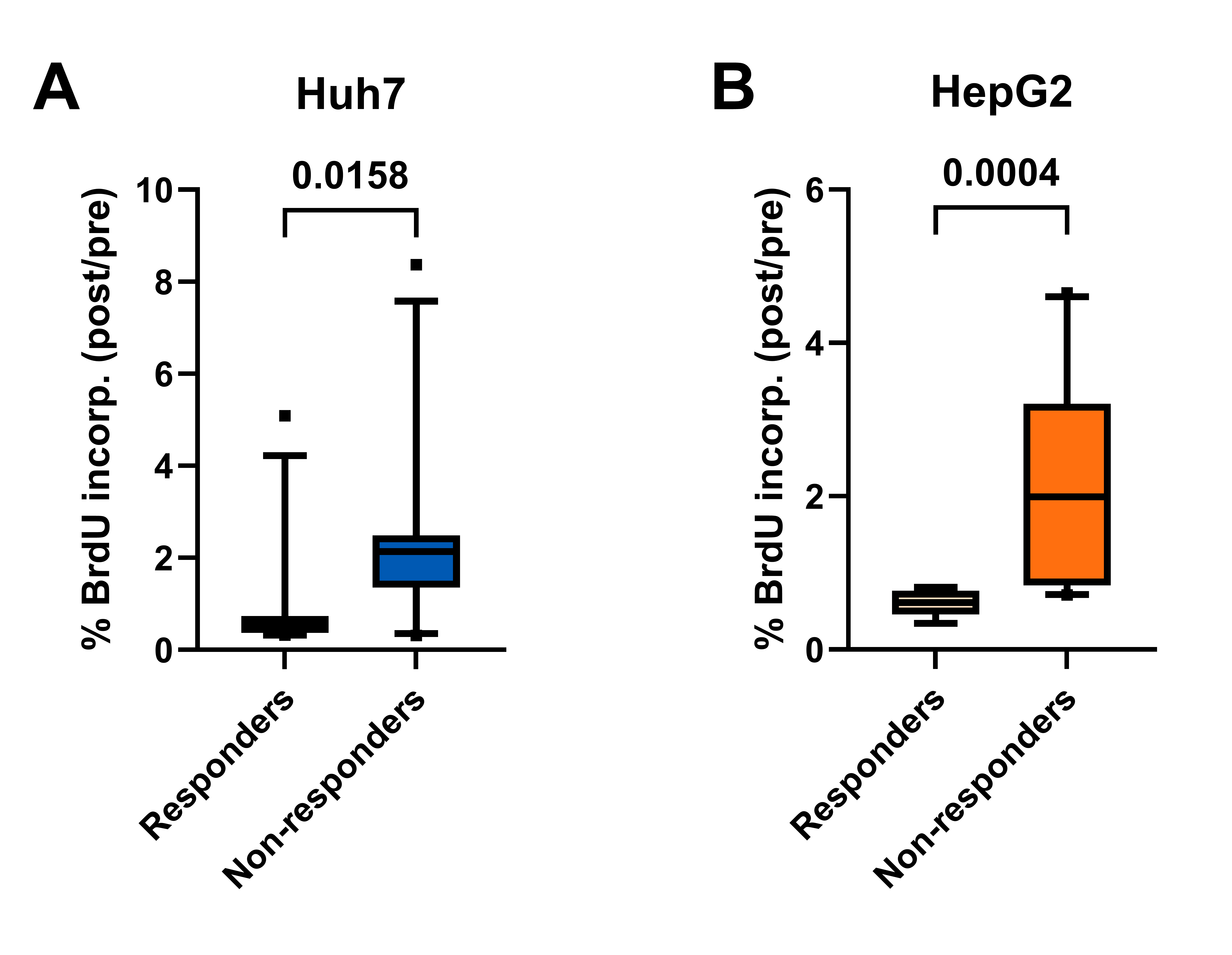

Supplement: Supplementary file 1 — Fig. S1. BrdU incorporation after serum incubation in responders and nonresponders. Fig. S2. Comparison of BrdU incorporation levels between responders and nonresponders. Fig. S3. FCS‐deprivation in Huh7 and HepG2 cells. Fig. S4. Time to systemic progression (TTSP) in accordance with BrdU incorporation for combined analysis of Huh7 and HepG2. Fig. S5. Heatmap illustrating the intensity of BrdU incorporation and plasma protein levels per patient. Table S1. Clinical and technical characteristics of the observed 23 HCC patients undergoing HDR‐BT. Table S2. Laboratory baseline parameters of the observed 23 HCC patients undergoing HDR‐BT. Table S3. (A) Absolute BrdU incorporation after serum incubation of Huh7. (B) Absolute BrdU incorporation after serum incubation of HepG2. [file MOL2-20-480-s001.zip › MOL2_70122_f2_SUPPL_FIG2.TIF.tif]

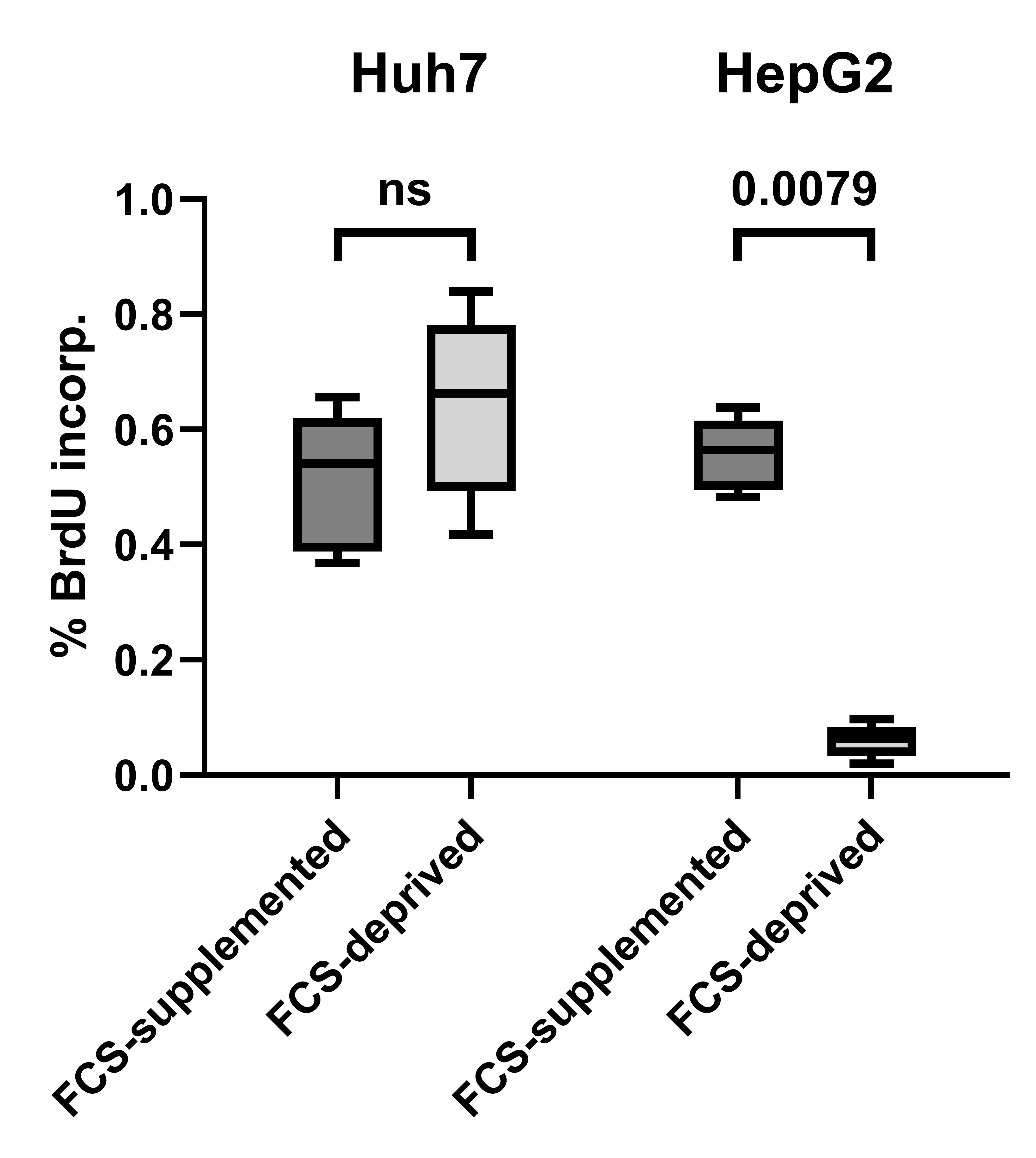

Supplement: Supplementary file 1 — Fig. S1. BrdU incorporation after serum incubation in responders and nonresponders. Fig. S2. Comparison of BrdU incorporation levels between responders and nonresponders. Fig. S3. FCS‐deprivation in Huh7 and HepG2 cells. Fig. S4. Time to systemic progression (TTSP) in accordance with BrdU incorporation for combined analysis of Huh7 and HepG2. Fig. S5. Heatmap illustrating the intensity of BrdU incorporation and plasma protein levels per patient. Table S1. Clinical and technical characteristics of the observed 23 HCC patients undergoing HDR‐BT. Table S2. Laboratory baseline parameters of the observed 23 HCC patients undergoing HDR‐BT. Table S3. (A) Absolute BrdU incorporation after serum incubation of Huh7. (B) Absolute BrdU incorporation after serum incubation of HepG2. [file MOL2-20-480-s001.zip › MOL2_70122_f3_SUPPL_FIG3.TIF.tif]

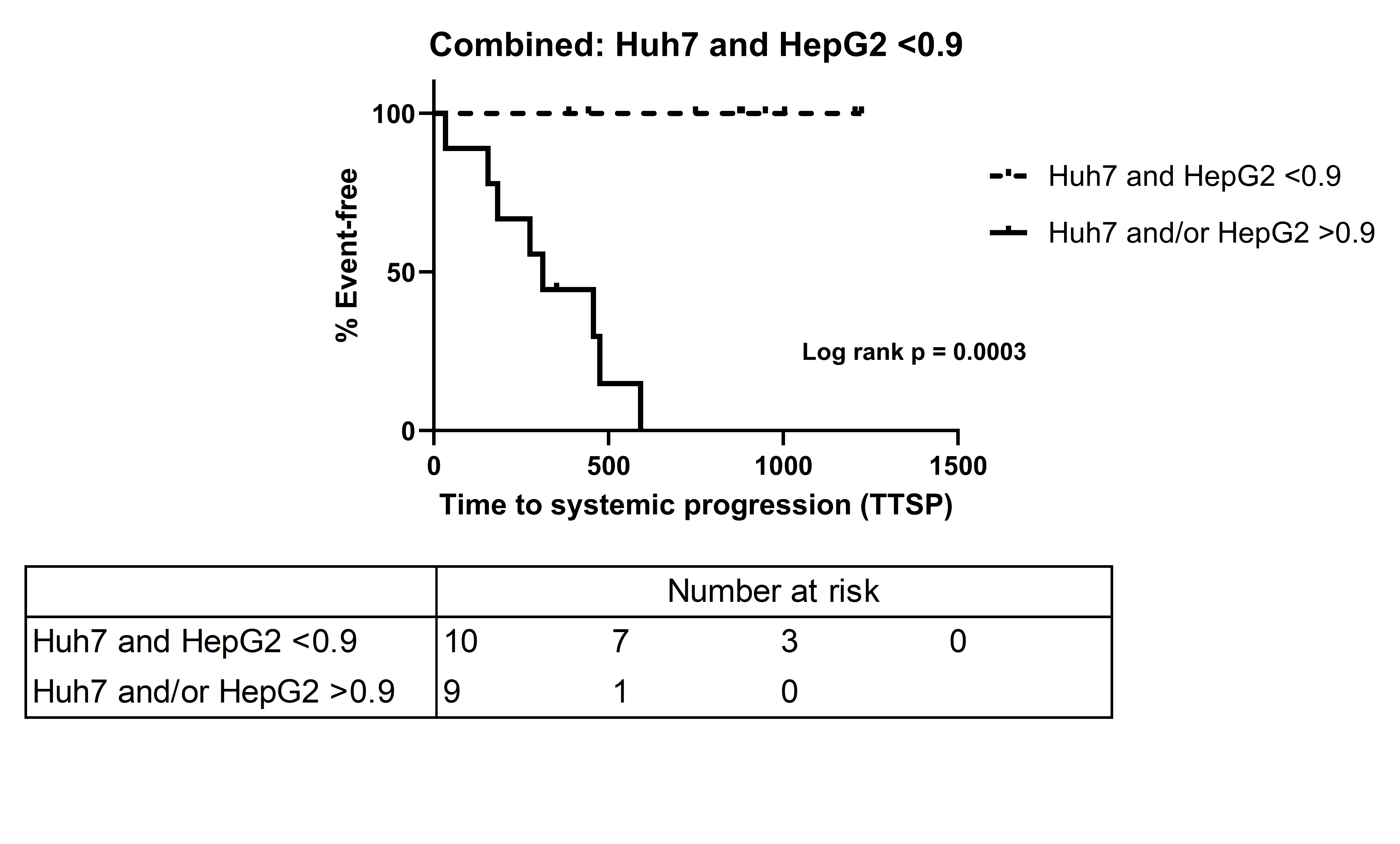

Supplement: Supplementary file 1 — Fig. S1. BrdU incorporation after serum incubation in responders and nonresponders. Fig. S2. Comparison of BrdU incorporation levels between responders and nonresponders. Fig. S3. FCS‐deprivation in Huh7 and HepG2 cells. Fig. S4. Time to systemic progression (TTSP) in accordance with BrdU incorporation for combined analysis of Huh7 and HepG2. Fig. S5. Heatmap illustrating the intensity of BrdU incorporation and plasma protein levels per patient. Table S1. Clinical and technical characteristics of the observed 23 HCC patients undergoing HDR‐BT. Table S2. Laboratory baseline parameters of the observed 23 HCC patients undergoing HDR‐BT. Table S3. (A) Absolute BrdU incorporation after serum incubation of Huh7. (B) Absolute BrdU incorporation after serum incubation of HepG2. [file MOL2-20-480-s001.zip › MOL2_70122_f4_SUPPL_FIG4.TIF.tif]

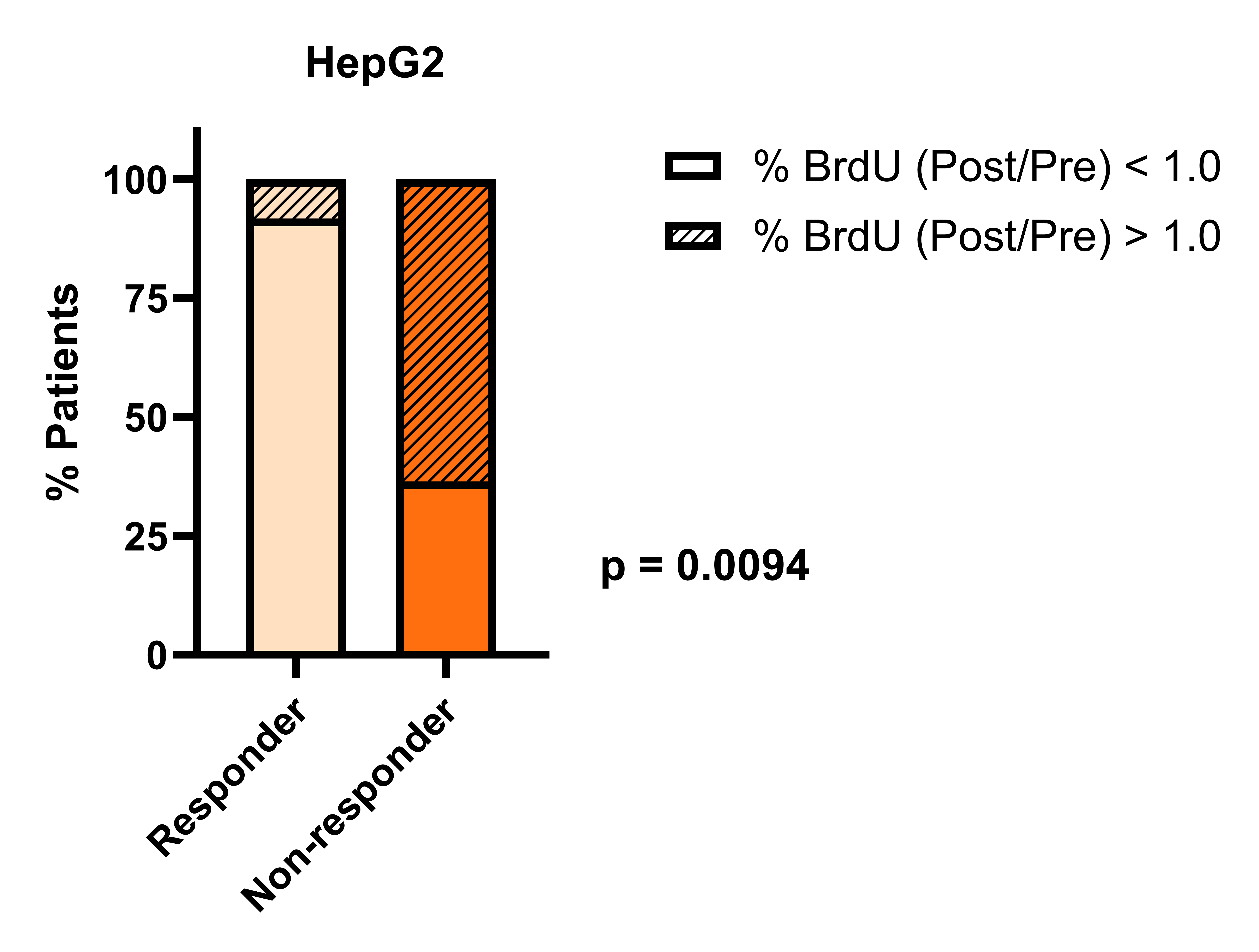

Supplement: Supplementary file 1 — Fig. S1. BrdU incorporation after serum incubation in responders and nonresponders. Fig. S2. Comparison of BrdU incorporation levels between responders and nonresponders. Fig. S3. FCS‐deprivation in Huh7 and HepG2 cells. Fig. S4. Time to systemic progression (TTSP) in accordance with BrdU incorporation for combined analysis of Huh7 and HepG2. Fig. S5. Heatmap illustrating the intensity of BrdU incorporation and plasma protein levels per patient. Table S1. Clinical and technical characteristics of the observed 23 HCC patients undergoing HDR‐BT. Table S2. Laboratory baseline parameters of the observed 23 HCC patients undergoing HDR‐BT. Table S3. (A) Absolute BrdU incorporation after serum incubation of Huh7. (B) Absolute BrdU incorporation after serum incubation of HepG2. [file MOL2-20-480-s001.zip › MOL2_70122_f4_SUPPL_FIG4B.TIF.tif]

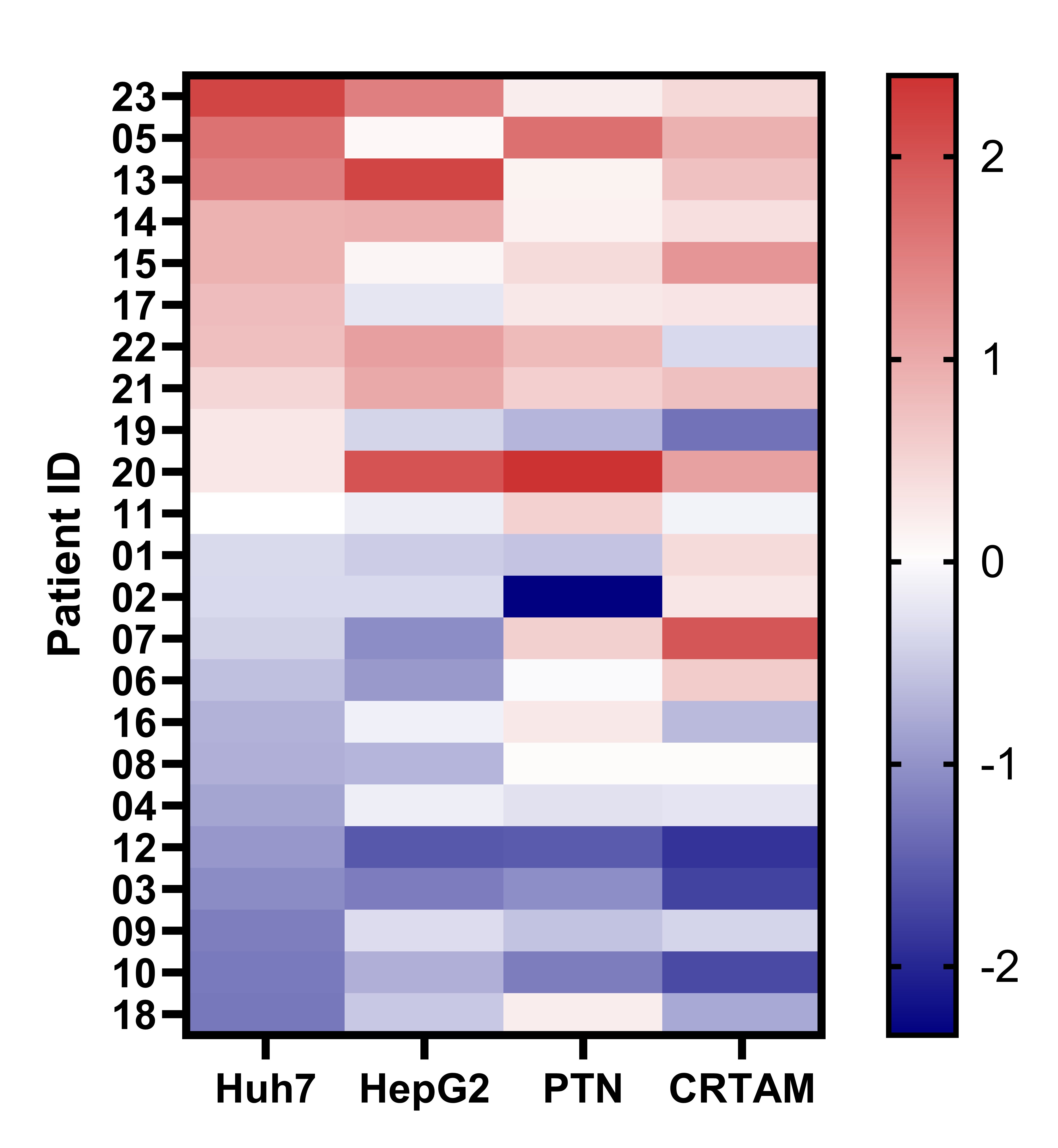

Supplement: Supplementary file 1 — Fig. S1. BrdU incorporation after serum incubation in responders and nonresponders. Fig. S2. Comparison of BrdU incorporation levels between responders and nonresponders. Fig. S3. FCS‐deprivation in Huh7 and HepG2 cells. Fig. S4. Time to systemic progression (TTSP) in accordance with BrdU incorporation for combined analysis of Huh7 and HepG2. Fig. S5. Heatmap illustrating the intensity of BrdU incorporation and plasma protein levels per patient. Table S1. Clinical and technical characteristics of the observed 23 HCC patients undergoing HDR‐BT. Table S2. Laboratory baseline parameters of the observed 23 HCC patients undergoing HDR‐BT. Table S3. (A) Absolute BrdU incorporation after serum incubation of Huh7. (B) Absolute BrdU incorporation after serum incubation of HepG2. [file MOL2-20-480-s001.zip › MOL2_70122_f5_SUPPL_FIG5.TIF.tif]
